# Supplementary material for: Safety and COVID-19 Symptoms in Individuals Recently Vaccinated with BCG: a Retrospective Cohort Study
Source: Cell Rep Med. 2020 Aug 5;1(5):100073. doi: 10.1016/j.xcrm.2020.100073 (PMC7405881; doi:10.1016/j.xcrm.2020.100073)
Supplement: Document S1. Figures S1–S6 [file mmc1.pdf]

Cell Reports Medicine, Volume *1*

Supplemental Information

Safety and COVID-19 Symptoms in Individuals Recently Vaccinated with BCG: a Retrospective Cohort Study

Simone J.C.F.M. Moorlag, Rosanne C. van Deuren, Cornelis H. van Werkhoven, Martin Jaeger, Priya Debisarun, Esther Taks, Vera P. Mourits, Valerie A.C.M. Koeken, L. Charlotte J. de Bree, Thijs ten Doesschate, Maartje C. Cleophas, Sanne Smeekens, Marije Oosting, Frank L. van de Veerdonk, Leo A.B. Joosten, Jaap ten Oever, Jos W.M. van der Meer, Nigel Curtis, Peter Aaby, Christine Stabell-Benn, Evangelos J. Giamarellos-Bourboulis, Marc Bonten, Reinout van Crevel, and Mihai G. Netea

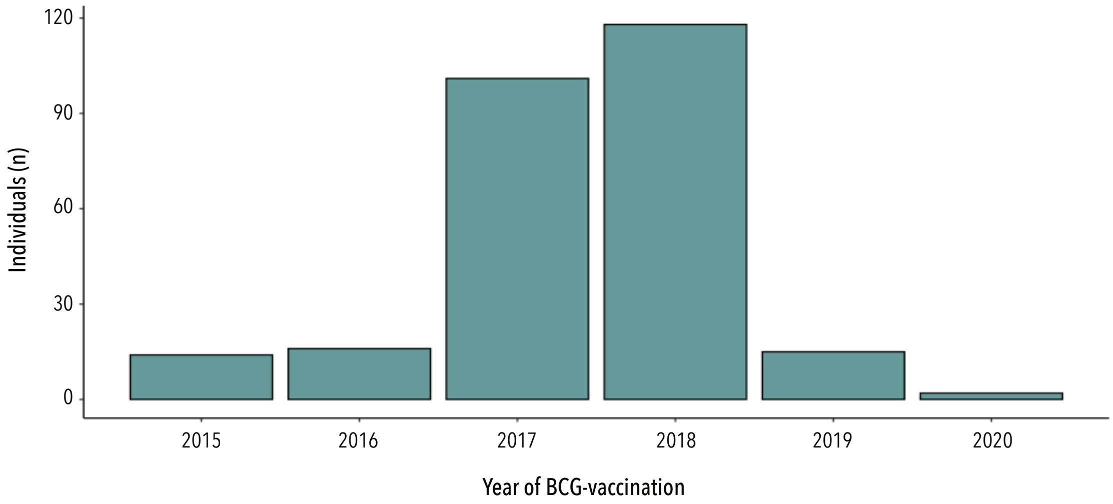

Figure S1 - Timing of BCG vaccination (n=266). Individuals vaccinated in 2017 and 2018 were vaccinated with BCG Intervax (Canada), in all other years individuals were vaccinated with BCG SSI (Denmark). Related to Table 1.

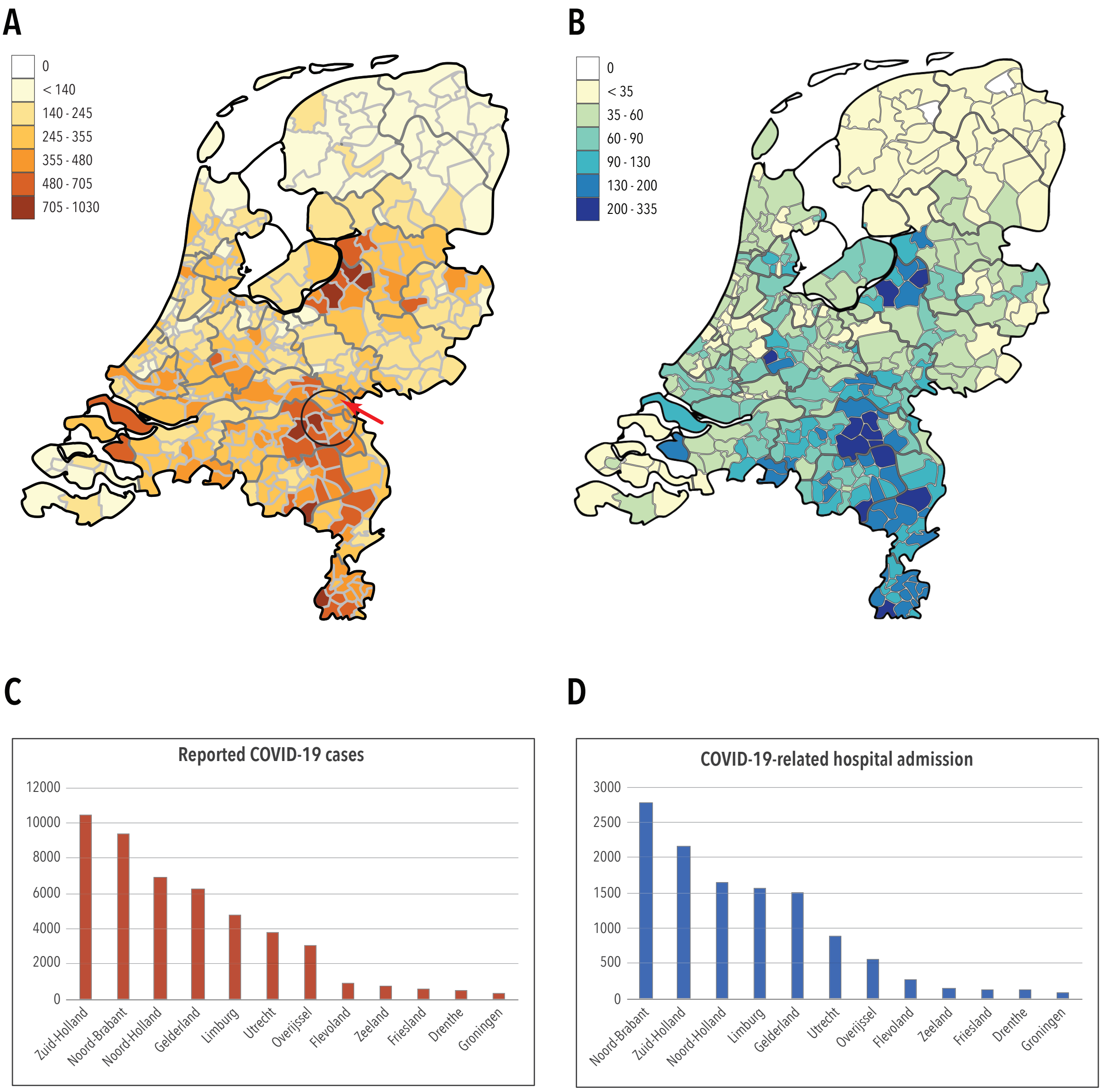

Figure S2 - Distribution of reported COVID-19 cases and COVID-19-related hospital admission in the Netherlands. (A) Reported COVID-19 cases in the Netherlands per 100000 inhabitants, as of June 8, 2020. The actual number of COVID-19 cases is higher as not all individuals are tested. Participants from all cohorts in this study were included at the Radboud University Medical Center (Radboudumc) in Nijmegen (red arrow). Most study participants live in the area indicated by the black circle. (B) Reported number of COVID-19 patients admitted to the hospital per 100000 inhabitants. (C) COVID-19 reported cases in the Netherlands as of June 8, 2020, by province. (D) COVID-19-related hospital admission in the Netherlands as of June 8, 2020, by province. Related to Figure 2.
Source: RIVM: Epidemiologische situatie COVID-19. Nederland. 08-06-2020.

A

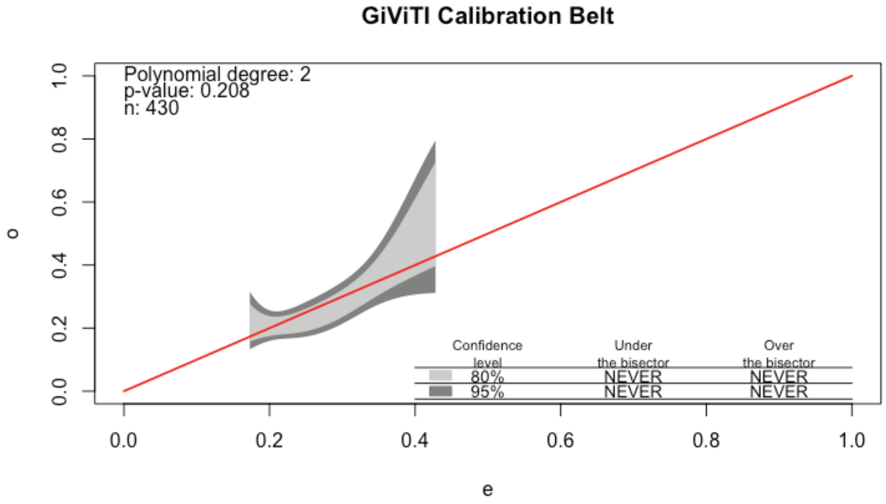

B

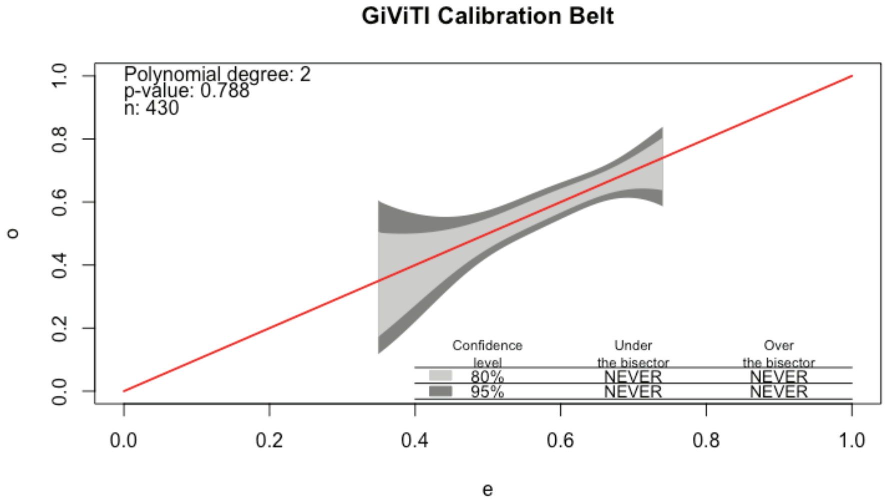

C

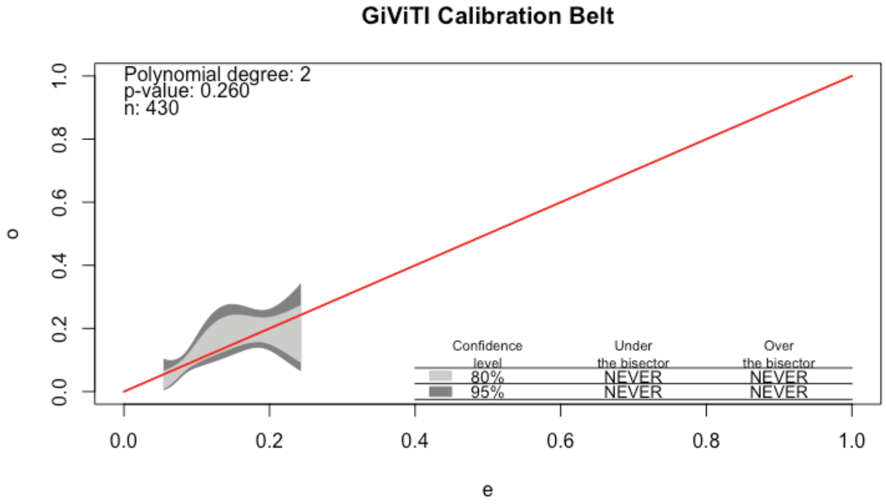

Figure S3 - Logistic Model fit. GivitICalibrationBelt-plots representing the model fit for the logistic models with ‘sickness’ (A), ‘any symptoms’ (B), and ‘extreme fatigue’ (C) as output variables. Related to Figure 2.

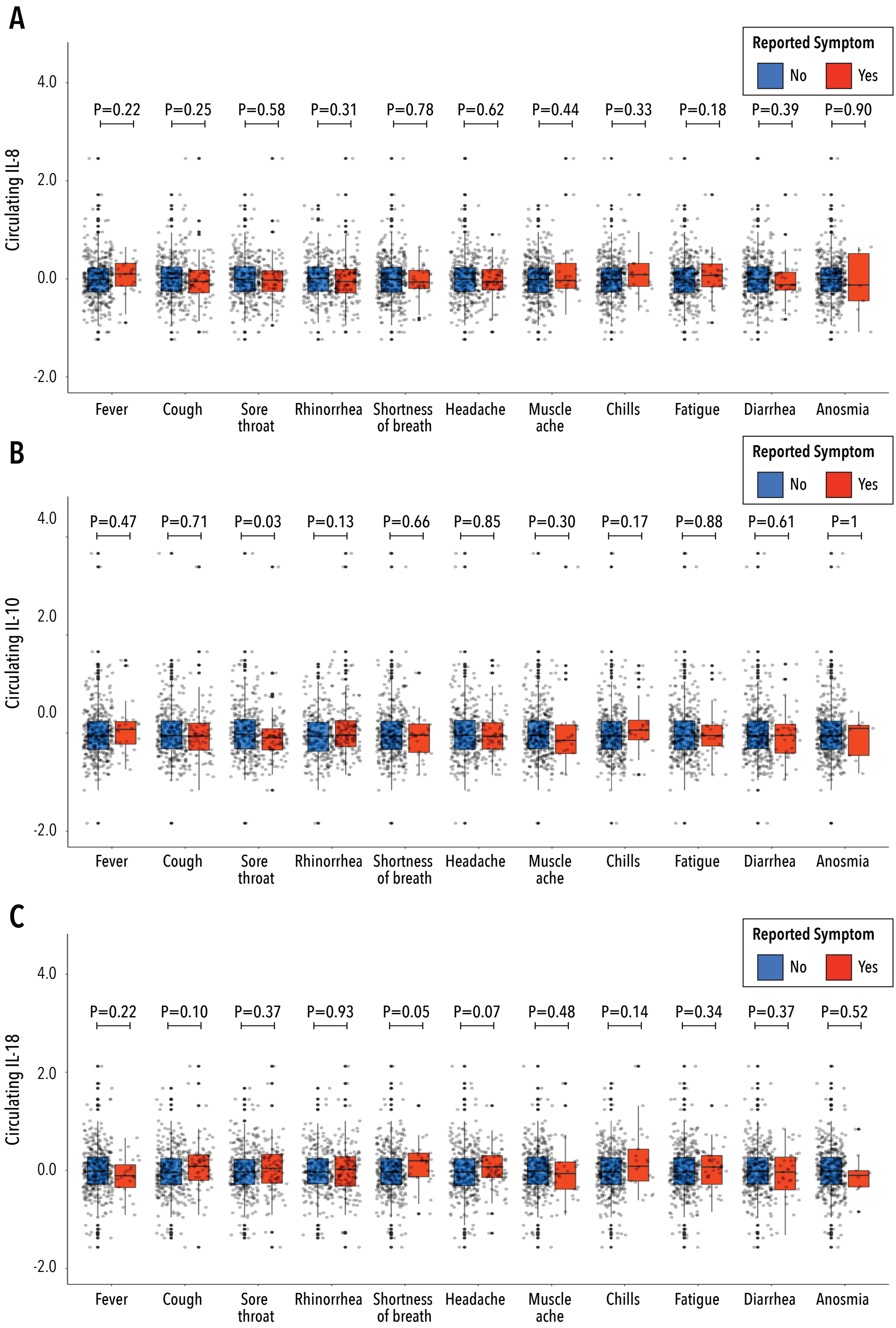

Figure S4 - Symptoms during the SARS-CoV-2 pandemic do not correlate with circulating immune parameters (n=392). Circulating concentrations of IL-8 (A), IL-10 (B) , and IL-18 (C) were normalized and measured on a log2-scale as normalized protein expression values. Differences in circulating immune parameters between individuals that reported a specific symptom were assessed using the Wilcoxon-rank sum test, and are annotated at the top of each plot. Related to Figure 3.

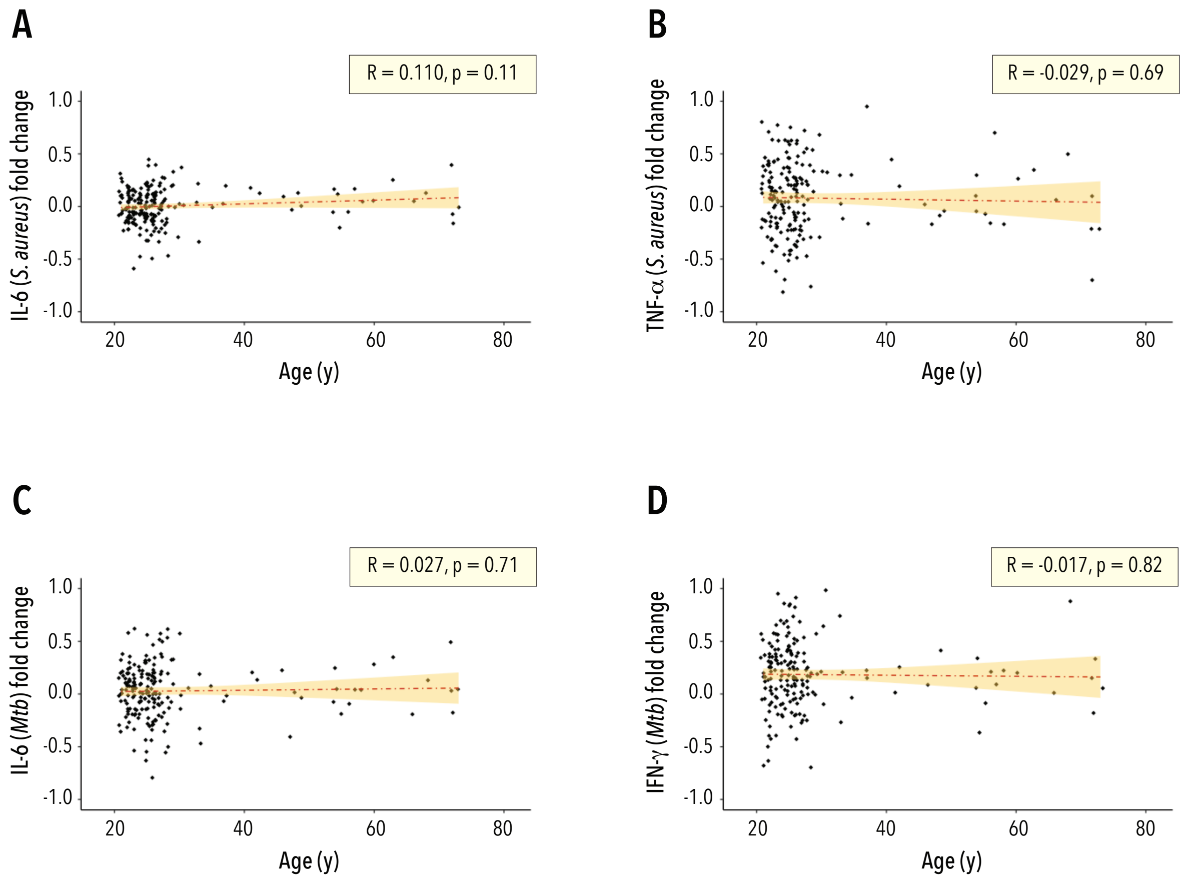

Figure S5 - Impact of age on innate memory responses induced by BCG vaccination. Log10-transformed fold changes of cytokine production upon stimulation is shown in correlation with age, with accompanying Pearson correlation and p-value. (A) IL-6 (*S. aureus*) n=195; (B) TNF-α (*S. aureus*) n=195; (C) IL-6 (*Mtb*) n=193; (D) IFN-γ (*Mtb*) n=189. Related to Figure 4.

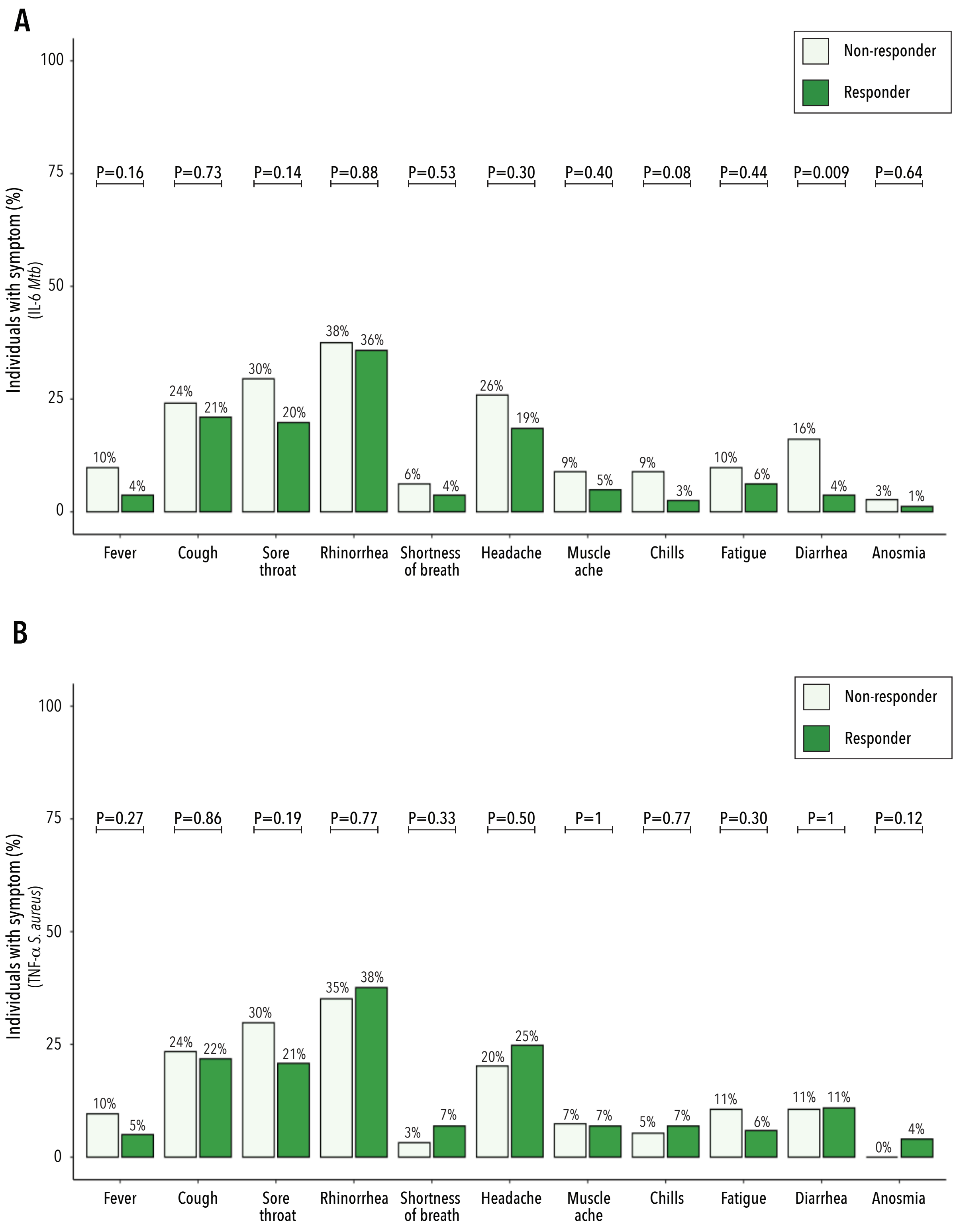

Figure S6 - Trained immunity responses correlate with self-reported symptoms during the SARS-CoV-2 pandemic. Fold change cytokine production of *Mtb*-induced IL-6 cytokine (A; n=193) and *S. aureus*-induced TNF-α cytokine (B; n=195) is shown in barplots for each reported symtpom, where individuals with a fold-change of 1.2 or higher were considered responders, and individuals with a fold change below 1.2 were considered non-responders. Percentages of non-responders (A; n=112, B; n=94) and responders (A; n=81, B; n=101) that reported symptoms are annotated above each bar, and statistical significance is annotated at the top of the plot (Fisher's Exact test). Related to Figure 4.
