# Supplementary material for: Safety and COVID-19 Symptoms in Individuals Recently Vaccinated with BCG: a Retrospective Cohort Study
Source: Cell Rep Med. 2020 Aug 5;1(5):100073. doi: 10.1016/j.xcrm.2020.100073 (PMC7405881; doi:10.1016/j.xcrm.2020.100073)
Supplement: Document S2. Article plus Supplemental Information [file mmc3.pdf]

Cell Reports
Medicine

Article

Safety and COVID-19 Symptoms in Individuals Recently Vaccinated with BCG: a Retrospective Cohort Study

Graphical Abstract

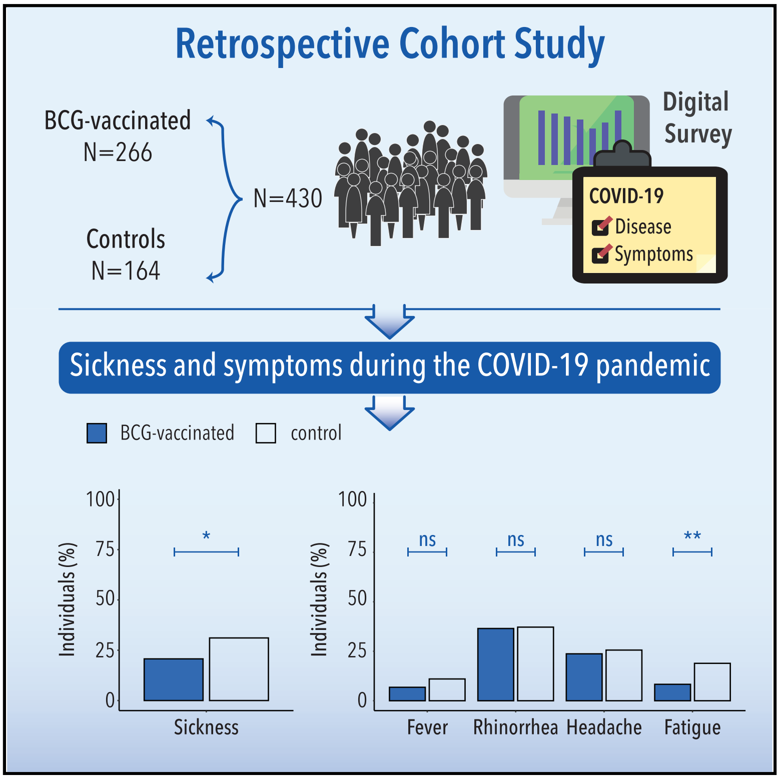

Highlights

- Recent BCG vaccination is safe during the COVID-19 pandemic
- BCG vaccination is not associated with symptoms of hyperinflammation
- BCG might be associated with reduced incidence of sickness and extreme fatigue
- Randomized trials of BCG vaccination for the prevention of COVID-19 are warranted

Authors

Simone J.C.F.M. Moorlag, Rosanne C. van Deuren, Cornelis H. van Werkhoven, ..., Marc Bonten, Reinout van Crevel, Mihai G. Netea

Correspondence

mihai.netea@radboudumc.nl

In Brief

Moorlag et al. show in a retrospective cohort study that recent BCG vaccination is safe and did not increase disease symptoms during the COVID-19 outbreak. Prospective randomized clinical trials are warranted to assess BCG vaccination effectiveness against SARS-CoV-2 infection.

Moorlag et al., 2020, Cell Reports Medicine *1*, 100073
August 25, 2020 © 2020 The Author(s).
https://doi.org/10.1016/j.xcrm.2020.100073

Cell Reports Medicine

CellPress OPEN ACCESS

Article

Safety and COVID-19 Symptoms in Individuals Recently Vaccinated with BCG: a Retrospective Cohort Study

Simone J.C.F.M. Moorlag,[1,12] Rosanne C. van Deuren,[1,2,12] Cornelis H. van Werkhoven,[3] Martin Jaeger,[1] Priya Debisarun,[1] Esther Taks,[1] Vera P. Mourits,[1] Valerie A.C.M. Koeken,[1] L. Charlotte J. de Bree,[1,6,7] Thijs ten Doesschate,[3] Maartje C. Cleophas,[1] Sanne Smeekens,[1] Marije Oosting,[1] Frank L. van de Veerdonk,[1] Leo A.B. Joosten,[1] Jaap ten Oever,[1] Jos W.M. van der Meer,[1] Nigel Curtis,[4] Peter Aaby,[5] Christine Stabell-Benn,[6,7] Evangelos J. Giamarellos-Bourboulis,[8,9] Marc Bonten,[10] Reinout van Crevel,[1] and Mihai G. Netea[1,11,13,*]

[1]Department of Internal Medicine and Radboud Center for Infectious Diseases (RCI), Radboud University Medical Center, Nijmegen, the Netherlands

[2]Department of Human Genetics, Radboud University Medical Center, Nijmegen, the Netherlands

[3]The Julius Center for Health Sciences and Primary Care, University Medical Center Utrecht, Utrecht University, Utrecht, the Netherlands

[4]Department of Paediatrics, The University of Melbourne; and Murdoch Children's Research Institute, Royal Children's Hospital Melbourne, Parkville, VIC 3052, Australia

[5]Bandim Health Project, Indepth Network, Bissau, Guinea-Bissau

[6]Danish Institute for Advanced Study, University of Southern Denmark, Denmark

[7]OPEN, Institute of Clinical Research, University of Southern Denmark/Odense University Hospital, Odense, Denmark

[8]4th Department of Internal Medicine, National and Kapodistrian University of Athens, Athens, Greece

[9]4th Department of Internal Medicine, ATTIKON University Hospital, Athens, Greece

[10]Department of Medical Microbiology, The Julius Center for Health Sciences and Primary Care, University Medical Center Utrecht, Utrecht University, Utrecht, the Netherlands

[11]Immunology and Metabolism, Life and Medical Sciences Institute (LIMES), University of Bonn, Bonn, Germany

[12]These authors contributed equally

[13]Lead Contact

*Correspondence: mihai.netea@radboudumc.nl

https://doi.org/10.1016/j.xcrm.2020.100073

SUMMARY

Bacille Calmette-Guérin (BCG) induces long-term boosting of innate immunity, termed trained immunity, and decreases susceptibility to respiratory tract infections. BCG vaccination trials for reducing SARS-CoV-2 infection are underway, but concerns have been raised regarding the potential harm of strong innate immune responses. To investigate the safety of BCG vaccination, we retrospectively assessed coronavirus disease 2019 (COVID-19) and related symptoms in three cohorts of healthy volunteers who either received BCG in the last 5 years or did not. BCG vaccination is not associated with increased incidence of symptoms during the COVID-19 outbreak in the Netherlands. Our data suggest that BCG vaccination might be associated with a decrease in the incidence of sickness during the COVID-19 pandemic (adjusted odds ratio [AOR] 0.58, $p < 0.05$), and lower incidence of extreme fatigue. In conclusion, recent BCG vaccination is safe, and large randomized trials are needed to reveal if BCG reduces the incidence and/or severity of SARS-CoV-2 infection.

INTRODUCTION

Coronavirus disease 2019 (COVID-19) is a respiratory tract infection caused by a new coronavirus named severe acute respiratory syndrome coronavirus-2 (SARS-CoV-2). COVID-19 varies from asymptomatic SARS-CoV-2 infection or mild upper respiratory tract infection in the majority of patients, to severe pneumonia with acute respiratory distress syndrome (ARDS) and respiratory insufficiency in a small but significant number of patients.[1] Since the disease was first described at the end of 2019 in Wuhan, China, SARS-CoV-2 has spread rapidly throughout the world and on March 11th, 2020 the World Health Organization (WHO) declared the coronavirus outbreak a pandemic.[2] The large number of patients, caused by the lack of immunity in the population, has put a heavy burden on the healthcare system. Despite aggressive containment measures, the number of infections is rising in many countries, with Europe and United States now being the hotspots of the epidemic,[3] while the number of cases in low-income countries will likely rise in the coming period.

It is expected that the infection will remain endemic in the population for years to come, with regular outbreaks when quarantine measures are relaxed and in the winter seasons. Vaccination would be the most optimal tool for infection prevention. Although more than 80 different initiatives around the world are trying to develop a disease-specific vaccine, it is likely that at least

This is an open access article under the CC BY license (http://creativecommons.org/licenses/by/4.0/).

1.5–2 years will be needed to produce an effective vaccine. Other measures to contain the infection are therefore urgently needed.

Bacille Calmette-Guérin (BCG) induces a general long-term boosting of innate immune mechanisms, also termed "trained immunity," mediated by an epigenetic, transcriptional, and functional reprogramming of innate immune cells such as monocytes, macrophages, and natural killer cells.[4] Epidemiological studies and randomized trials have shown that BCG vaccination reduces all-cause infant mortality by diminishing deaths due to infections other than tuberculosis.[5–8] The observation that BCG vaccination could protect against viral infection was strengthened by a study in Guinea-Bissau showing that BCG reduced the incidence of respiratory syncytial virus infection.[9] Protective effects of BCG vaccination on respiratory tract infections have been reported in elderly in Indonesia[10] and Japan.[11] In addition, we have previously shown that BCG can decrease viral replication and viral load in a model of human experimental viral infection with attenuated yellow fever vaccine virus.[12]

Based on these data, it has been hypothesized that BCG vaccination may also have beneficial effects against SARS-CoV-2 infection.[13] Several clinical trials to assess the effect of BCG vaccination on COVID-19 are underway (https://clinicaltrials.gov, e.g., NCT04417335, NCT04328441 and NCT04327206). However, concerns have been raised regarding the potential harm that would be caused by an excessive inflammatory response induced by BCG in some patients with COVID-19[13]: indeed, deleterious cytokine production has been reported to lead to ARDS and death in COVID-19 patients on the intensive care.[1,14] Although no data have been reported regarding a potential deleterious effect of BCG vaccination, it is important to assess the direct safety profile of BCG vaccination during the COVID-19 pandemic, considering that more than 10 trials are either underway or planned in different countries. We therefore compared disease and symptoms during the COVID-19 outbreak in volunteers who were either recently vaccinated (within the last 5 years) with BCG or did not receive a BCG vaccine. We report that recent BCG vaccination is safe in the COVID-19 pandemic in the populations studied, and based on that, we argue that large randomized trials of BCG vaccination for the prevention of SARS-CoV-2 infections are warranted.

RESULTS

To investigate the safety of recent BCG vaccination in the SARS-CoV-2 pandemic, we retrospectively compared in parallel three similar cohorts of volunteers who were either vaccinated with BCG in the last 5 years (266 volunteers) or had never received a BCG vaccine (164 volunteers) (Figure 1). These cohorts are part of the Human Functional Genomics Project (HFGP) (http://www.humanfunctionalgenomics.org) and comprise healthy volunteers of Western-European ancestry. The majority of individuals who received a BCG vaccine were vaccinated with BCG between April 2017 and June 2018 (Figure S1). The first case that tested positive for SARS-CoV-2 was detected in the Netherlands on February 27th 2020, and this was followed by a dramatic increase in cases in the weeks thereafter, with a peak of reported COVID-19 cases in the first half of April. Therefore, we retrospectively collected information about COVID-19-related disease and symptoms that occurred in the period between February 27th and April 30th 2020. These data, as well as other metadata including recent travel history and contact with SARS-CoV-2-infected individuals, was collected via two digital surveys (see Table S1 for an overview of survey questions). In the first survey, we collected demographic characteristics and information on the first 4 weeks of the epidemic in the Netherlands (time period between February 27th and March 31st). Participants that completed the first survey were sent a second survey in which we queried participants about the time period between April 1st and April 30th. Participants that completed both surveys were included in the analysis, while individuals who received a BCG vaccination within the time period assessed were excluded. Baseline characteristics of the BCG-vaccinated individuals and controls that completed both surveys are summarized in Table 1. The two groups were similar in sex distribution, BMI, and underlying chronic conditions (including lung disease and allergic rhinitis). However, the proportion of individuals in the BCG-vaccinated group age 60 years and older was lower compared to the control group (4.5% versus 12.8%, $p < 0.01$). The median age of BCG-vaccinated individuals was 25 years (age range, 21–73 years) compared to a median age of 30 years (age range, 23–80 years) in the control group. On the other hand, BCG-vaccinated individuals had more often been in contact with SARS-CoV-2 infected individuals compared to the control group (36.8% versus 23.8%, $p < 0.01$) and were more often healthcare workers compared to the control group (49.2% versus 37.2%, $p < 0.05$), which could be an indication of an increased risk of exposure to and infection with SARS-CoV-2 in the BCG-vaccinated group.[15,16] We corrected for all these differences in a statistical model of logistical regression.

BCG Vaccination Does Not Increase Reported SARS-CoV-2-Related Disease or Hospital Admission

To investigate the safety profile of BCG, we first compared SARS-CoV-2-related disease between the BCG-vaccinated and control group based on the survey results. No differences in the number of cases diagnosed with COVID-19 (by PCR or by a physician based on clinical symptoms) were observed between the BCG and control groups (1.5% versus 1.2%, $p = 1$) (Figure 2A). Importantly, none of the BCG-vaccinated (or control) individuals reported admission to hospital, suggesting that BCG vaccination is not associated with increased risk of hospitalization during the SARS-CoV-2 pandemic in this population (Figure 2B). Serological surveys in the Netherlands show that between 5.5%–10.0% of the population has been infected, which means that at least 14–27 individuals in the BCG group have been infected during the first pandemic wave. Despite of that, none of the volunteers needed hospitalization. Collectively, these findings provide reassurance that recent BCG vaccination is safe and not associated with an increase in COVID-19 or an increase in severity of disease in a population of healthy individuals.

BCG Vaccination Is Not Associated with Increased Sickness and Symptoms during the SARS-CoV-2 Pandemic

In the time period assessed, the testing policy in the Netherlands focused on healthcare workers caring for vulnerable groups. This

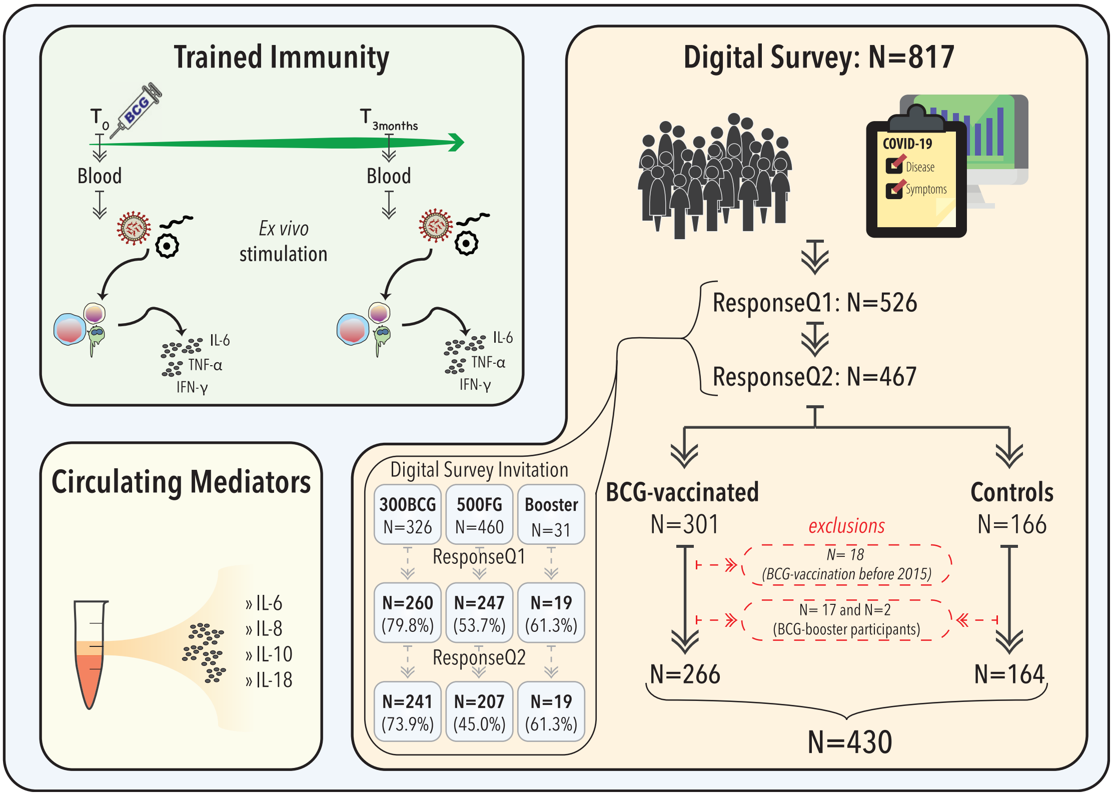

Figure 1. Schematic Overview of the Study

The dataset consists of 430 adult volunteers of which 266 volunteers received the BCG vaccine in the last 5 years and 164 individuals did not receive the BCG vaccine (control group). Information about disease and any symptoms between February 27th to April 30th 2020, as well as other metadata including recent travel history and previous contact with SARS-CoV-2 infected individuals were collected via two digital surveys that were sent to participants of the 300BCG, 500FG, and BCG-booster cohorts. Participants that received a BCG vaccination before 2015 and participants from the BCG booster cohort that received a BCG vaccine between February 27th and April 30th were excluded from analysis. Circulating immune parameters were assessed in individuals from the 300BCG and 500FG cohorts. The BCG-induced trained immunity response was assessed in the 300BCG cohort by comparing cytokine levels upon *ex vivo* stimulation of peripheral blood mononuclear cells (PBMCs) 3 months after BCG vaccination to the levels before vaccination. Annotation: ResponseQ1 = response survey 1, containing information on disease and symptoms between February 27th to March 31st 2020; ResponseQ2 = response survey 2, containing information on disease and symptoms between April 1st to April 30th 2020. For information on timing of BCG vaccination see Figure S1.

means that individuals who felt sick or experienced symptoms as a result of a SARS-CoV-2 infection, but were not working in healthcare, were unlikely to be tested and have not all been seen by a physician. As a result, the actual number of COVID-19 cases in the Netherlands is higher than the number of documented cases.[17] Furthermore, the coronavirus did not equally spread throughout the Netherlands, resulting in significant regional differences in the number of COVID-19 cases.[18] The large majority of the individuals that participated in this study are either residents of the province Noord-Brabant, the epicenter of the COVID-19 outbreak in the Netherlands, or live in Gelderland, the province that borders Noord-Brabant. These regions are among the top provinces with the highest number of COVID-19 registered cases and COVID-19-related hospital admission in the country (Figure S2). For these reasons, it is possible that a significant number of participants in our cohorts was not tested for COVID-19, but did experience symptoms as a result of SARS-CoV-2 infection. In order to assess safety of BCG vaccination, we therefore next assessed potential differences in self-reported sickness and symptoms in the period between February 27th and April 30th 2020 in BCG-vaccinated individuals and controls. Sickness was defined as any illness that led to absenteeism from work or prevented the individual from performing activities outside the house.[19] Without adjusting for confounders, the incidence of self-reported sickness was not increased in BCG-vaccinated individuals. If anything, the incidence of sickness appeared to be significantly lower in the BCG-vaccinated group as compared to the control group (20.7% versus 31.1%, crude odds ratio 0.58, $p < 0.05$) (Figure 2C). No significant difference was observed in the mean number of days of sickness (3.8 versus 5.5 days in the control group, $p = 0.16$) (Figure 2D). However, various risk factors have been reported to be associated with SARS-CoV-2 infection, including age, underlying chronic conditions, and exposure to SARS-CoV-2-positive cases.[20] To account for possible covariates that might affect the correlation between BCG vaccination status and self-reported sickness, we created a logistic model with as output variable “self-reported sickness” and as predictor

Table 1. Baseline Characteristics of BCG-Vaccinated Individuals and Controls

Variable	BCG-Vaccinated (n = 266)	Control (n = 164)	p Value[a]
Male sex, n (%)	110 (41.4)	63 (38.4)	0.62
Age >60 years, n (%)	12 (4.5)	21 (12.8)	<0.01
BMI, mean (SD)	22.8 (2.7)	23.5 (3.4)	0.05
Presence of underlying chronic condition, n (%)	21 (7.9)	19 (11.6)	0.27
Heart disease, n (%)	2 (0.8)	2 (1.2)	0.64
High blood pressure, n (%)	4 (1.5)	7 (4.3)	0.11
Diabetes mellitus, n (%)	3 (1.1)	0 (0.0)	N/A
Lung disease (asthma/COPD), n (%)	11 (4.1)	10 (6.1)	0.37
Kidney disease, n (%)	4 (1.5)	2 (1.2)	1
Allergic rhinitis, n (%)	63 (23.7)	46 (28.0)	0.37
Employed, n (%)	208 (78.2)	134 (81.7)	0.45
College degree, n (%)	139 (52.3)	79 (48.2)	0.61
Any international travel January 1st–March 31st, no. individuals traveled (%)	118 (44.4)	64 (39.0)	0.32
Known contact with infected person, n (%)	98 (36.8)	39 (23.8)	<0.01
Works in healthcare, n (%)	131 (49.2)	61 (37.2)	<0.05

BCG, Bacille Calmette-Guérin vaccine; SD, standard deviation; BMI, body mass index; COPD, chronic obstructive pulmonary disease; No., number of.

[a]Chi-square test of independence was used for categorical variables. Wilcoxon rank-sum test was used for continuous variables. Fisher's exact test was used for the comparison of underlying conditions, employment status, and the variable college degree.

variables BCG vaccination, age, underlying chronic condition (none versus one or more), healthcare-work (yes versus no), known contact with infected person (yes versus no), and any international travel between January 1st and March 31st 2020 (yes versus no). The logistic model fit was appropriate (p = 0.21, Figure S3) and revealed an adjusted odds ratio (AOR) for BCG vaccination of 0.58 ($p < 0.05$), supporting our previous observation of less sickness in BCG-vaccinated individuals as compared to controls.

Similar to the incidence of reported sickness, the incidence of reporting at least one symptom was significantly lower in the BCG-vaccinated group as compared to the control group after adjusting for confounders (AOR 0.65, $p < 0.05$, model fit p = 0.79) (Figures 2E and S3). The most common reported symptoms were rhinorrhea and sore throat, followed by cough and headache (Figure 2F). The incidence of various reported COVID-19-related symptoms, including fever, cough, sore throat, shortness of breath, headache, and muscle ache was not significantly different between the BCG-vaccinated group and the control group. However, the incidence of extreme fatigue, a common symptom of COVID-19, was in the BCG-vaccinated group less than half of that in the control group (8.3% versus 18.9%, AOR 0.37, $p < 0.01$, model fit p = 0.26). Taken together, we observed less sickness and extreme fatigue in the BCG-vaccinated group, indicating that BCG vaccination is safe during the COVID-19 pandemic.

Association of Self-Reported Sickness and Symptoms during the SARS-CoV-2 Pandemic with Circulating Immune Parameters

To explore whether variation in baseline immune status correlates with the incidence of self-reported sickness, we compared the concentrations of the circulating cytokines interleukin (IL)-6, IL-8, IL-10, and IL-18 between individuals who had been sick and those who had not been sick. Measurements were performed on plasma of individuals from the 500FG and 300BCG cohorts that was obtained upon inclusion (2013/2014 500FG, 2017/2018 300BCG, n = 392). Similar concentrations of IL-6, IL-8, IL-10, and IL-18 were observed between the two groups (Figure 3A). In line with these findings, no differences were observed in circulating cytokines for any of the symptoms (Figures 3B and S4).

BCG-Responsiveness for Trained Immunity Is Not Associated with Sickness and Symptoms during the COVID-19 Pandemic

Previous studies have indicated that BCG might protect against viral infections through the induction of a long-lasting enhanced cytokine production capacity associated with trained immunity.[12,21] However, large inter-individual variation exists in the ability to build trained immunity responses, and this may be caused by host factors (both genetic and non-genetic)[22–24] and factors related to the microorganisms. As a result, differences in the magnitude of the trained immunity response may affect the off-target protective effects of BCG vaccination. In order to assess a potential difference in the incidence of sickness and symptoms during the COVID-19 outbreak between individuals with strong immune memory responses and individuals with lower trained immunity responses, we investigated the variability of immune memory responses in BCG-vaccinated individuals from the 300BCG cohort. Peripheral blood mononuclear cells (PBMCs) from participants were stimulated *ex vivo* with *Mycobacterium tuberculosis* (*M. tuberculosis* or *Mtb*) H37Rv (specific stimulus) or *Staphylococcus aureus* (*S. aureus*) (heterologous stimulus) before and 3 months after BCG vaccination. Cytokine production was measured in 24 h (IL-6 and tumor necrosis factor alpha [TNF-α]) and 7 days (interferon [IFN]-γ) supernatants. The magnitude of the trained immunity (innate immune memory) response was assessed by the fold change in cytokine production (IL-6 and TNF-α) 3 months after BCG vaccination as compared to levels before vaccination. The specific (adaptive) immune memory response was assessed by the fold change in IFN-γ production upon stimulation with *M. tuberculosis*. Consistent with previous studies, we observed a significant increase in cytokine production upon BCG vaccination as well as a high degree of inter-individual variability in the induction of BCG-induced immune memory responses (Figure 4A). Of note, age showed no correlation with the induction of trained immunity responses, indicating sustained trained immunity responses in the elderly (Figure S5).

To assess whether the ability to build a trained immunity response following BCG vaccination was associated with

CellPress
OPEN ACCESS

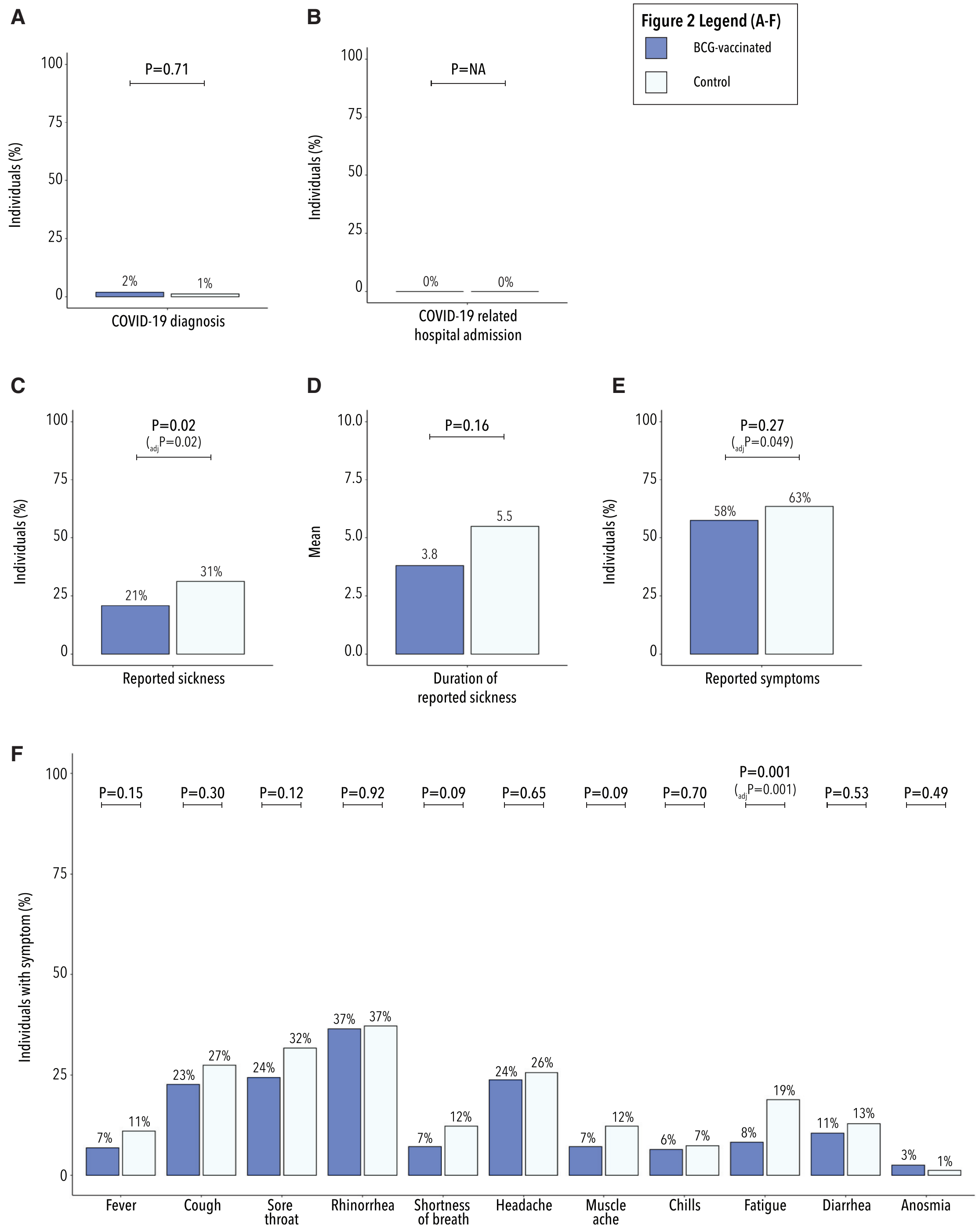

Figure 2. BCG Vaccination Is Not Associated with Increased Disease or Severity of COVID-19-Related Symptoms

(A) Percentage of participants with reported positive COVID-19 diagnosis (diagnosis made by PCR/physician based on clinical symptoms) in the BCG-vaccinated (n = 266) and control group (n = 164) (p = Fisher's exact test p value).

(B) Percentage of SARS-CoV-2-related hospital admission in the BCG-vaccinated (n = 266) and control group (n = 164).

(C) Percentage of self-reported sickness between February 27th and April 30th 2020 in the BCG-vaccinated (n = 266) and control group (n = 164) (p = chi-square test p value; $_{adj}$p = logistic regression adjusted p value).

(D) The average number of reported days of sickness in the BCG-vaccinated and control group (n = 106, p = Wilcoxon rank-sum test p value).

(E) Percentage of individuals that reported any symptom in the BCG-vaccinated (n = 266) and control group (n = 164) (p = chi-square test p value).

(F) Percentage of individuals that reported the indicated specific symptom between February 27th and April 30th 2020 in the BCG-vaccinated (n = 266) and control group (n = 164) (p = chi-square test p value).

See also Figure S2.

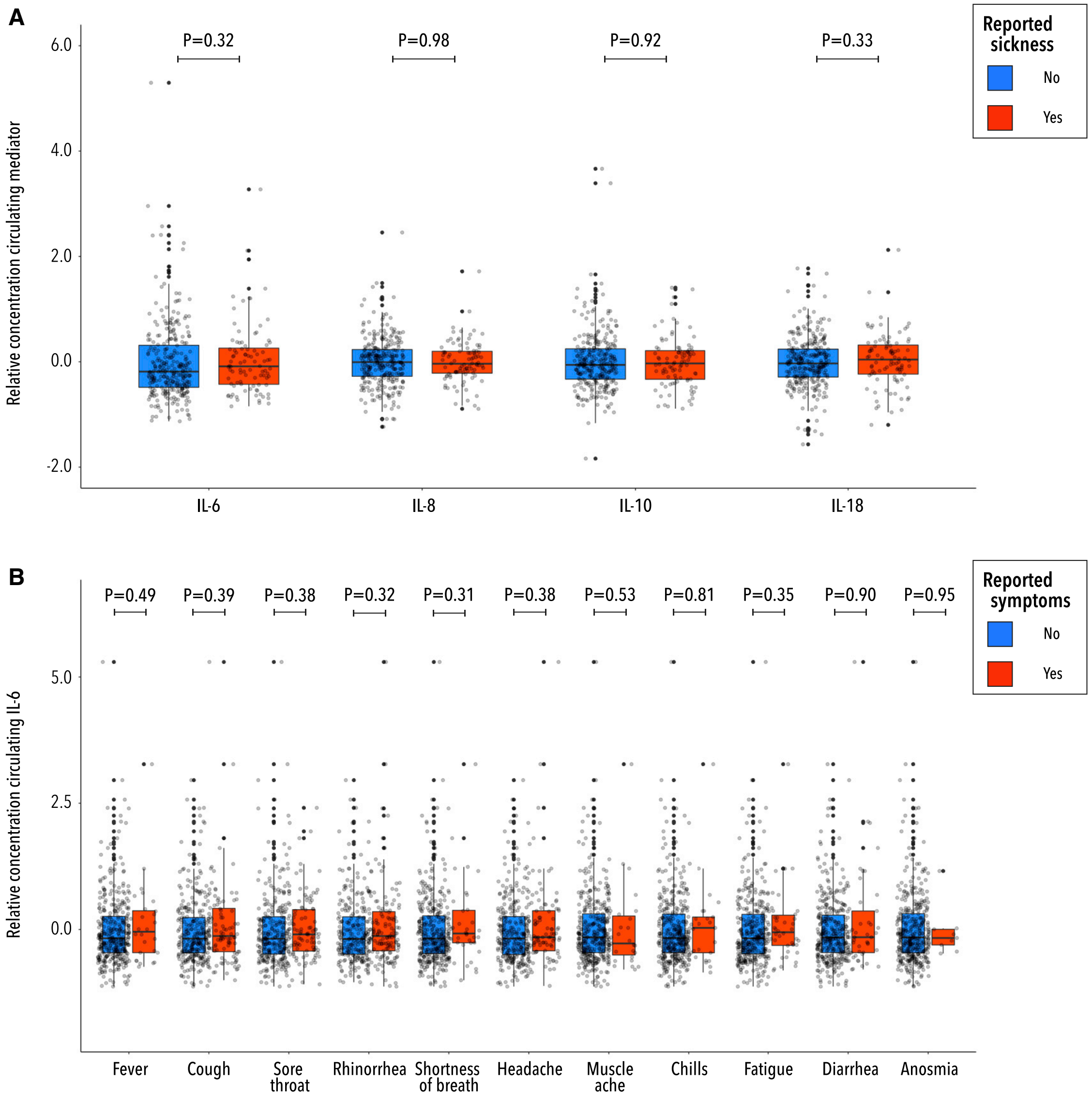

Figure 3. Self-Reported Sickness and Symptoms during the SARS-CoV-2 Pandemic Do Not Correlate with Circulating Immune Parameters
Circulating concentrations of IL-6, IL-8, IL-10, and IL-18 were assessed before BCG vaccination, normalized, and measured on a log2-scale as normalized protein expression values. Differences between self-reported sickness (A) or symptoms (B) and circulating immune parameters were assessed (n = 392; p = Wilcoxon rank-sum test p value). Boxes represent middle 50% of the data with a center line for the median, the lines extending the boxes roughly represent the 95% confidence interval for comparing medians, outliers are shown as separate dots.
See also Figure S4.

recent self-reported sickness and symptoms, individuals were divided into responders (based on fold change $\geq$1.2; *S. aureus*-induced IL-6, n = 67; *Mtb*-induced IL-6, n = 81; and *S. aureus*-induced TNF-α, n = 101) and non-responders (fold change <1.2; *S. aureus*-induced IL-6, n = 128; *Mtb*-induced IL-6, n = 112; and *S. aureus*-induced TNF-α, n = 94). The incidence of self-reported sickness (Figure 4B) as well as the incidence of symptoms (Figures 4C, 4D, and S6) was not significantly different between responders and non-responders, indicating that a strong trained immunity profile is not associated with increased sickness or severity of symptoms during the COVID-19 pandemic.

Aside from the correlation with the trained immunity response, the correlation of the specific (adaptive) immune memory response, sickness, and symptoms was determined by similarly dividing individuals into responders (based on fold change $\geq$1.5; *Mtb*-induced IFN-γ, n = 96) and non-responders (fold change <1.5; *Mtb*-induced IFN-γ, n = 93). Similar to our previous findings, no difference was observed in the incidence of sickness (Figure 4E) or reported symptoms (Figures 4F and 4G) between individuals with a strong specific immune memory response and those with weak responses. These data collectively suggest that individuals with strong BCG-induced immune memory responses do not show an increase in sickness and symptoms

CellPress
OPEN ACCESS

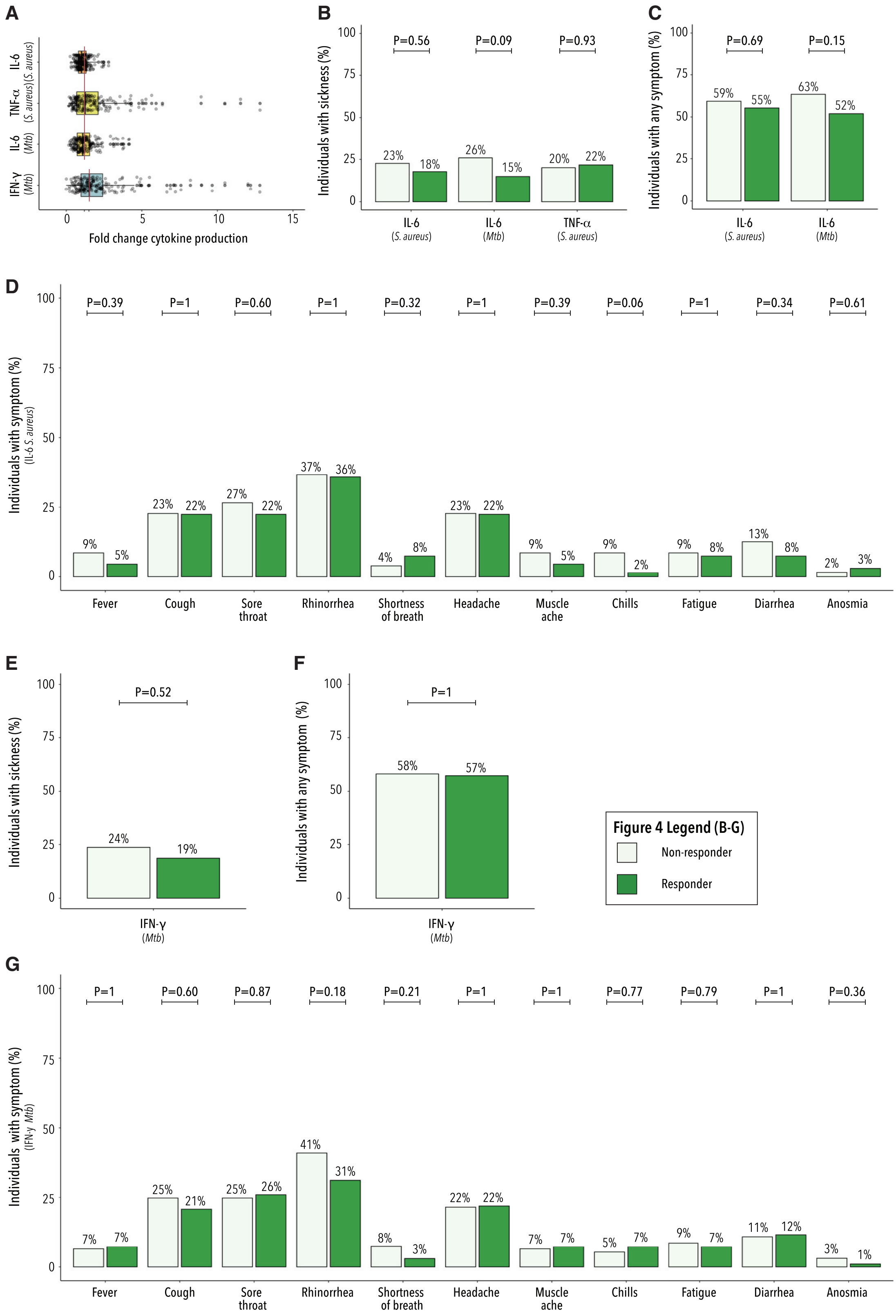

(legend on next page)

as compared to individuals showing weaker responses, suggesting that BCG vaccination could also be considered safe in the subgroup of individuals that display a strong immune memory profile upon BCG vaccination.

Finally, we assessed the BCG safety profile based on the results from the first digital survey, representing the first 4 weeks of the COVID-19 outbreak in the Netherlands (time period between February 27th and March 31st), to which more individuals responded. In total, 526 individuals completed the first survey. After exclusion of 20 individuals that received the BCG vaccine before 2015, the dataset consisted of 309 BCG-vaccinated individuals and 197 controls. Similar to the data analyzed for the time period between February 27th and April 30th, BCG vaccination was not associated with increased disease or severity of symptoms in the first 4 weeks of the COVID-19 pandemic in the Netherlands (Figure 5).

DISCUSSION

In the present study, we investigated whether a recent BCG vaccination is safe during the COVID-19 pandemic, and thus poses no significant risk if investigated in large clinical trials in populations at risk. A prophylactic approach using BCG vaccination against SARS-CoV-2 infection has recently been proposed due to the known beneficial off-target effects of BCG against other infections.[13,25–27] Importantly, however, an overwhelming inflammatory reaction has been described to contribute to severity and mortality in some patients with COVID-19,[14] which raised concerns that BCG vaccination, by enhancing innate immune responses, may have deleterious effects. In this study, we show that recent BCG vaccination is not associated with increased disease or severity of symptoms during the COVID-19 pandemic. If anything, BCG vaccination might be associated with a decrease in the incidence of sickness during the COVID-19 pandemic, as well as lower incidence of extreme fatigue, although these data need to be interpreted with caution considering the retrospective nature of the study.

Various clinical trials have shown that BCG vaccination strongly reduces all cause neonatal mortality, an effect that appeared to be due to protection against unrelated infectious agents, resulting in fewer cases of respiratory tract infections and neonatal sepsis.[5,8] These clinical trials have been complemented by experimental studies: Spencer et al.[28] showed that BCG reduced viral titers of influenza A virus in mice, an effect dependent on macrophages, while BCG vaccination also protected from herpes simplex virus type 2 in a controlled murine model with newborn mice.[29] We have demonstrated that epigenetic and functional reprogramming of monocytes[22] and their precursors in the bone marrow[30] leads to increased cytokine production capacity and antimicrobial activity,[31] which has been termed trained immunity.[32] Subsequently, BCG-induced trained immunity showed protective effects in a model of controlled experimental viral infection in humans.[12]

These data led to the hypothesis that BCG vaccination may also help against infection with SARS-CoV-2, and several large clinical trials are currently underway. Epidemiological studies seem to support this hypothesis by reporting lower rates of infection and mortality in countries with a childhood BCG vaccination program[25,33–35]; however, these studies are prone to biases[36] and only address the long-term effect of BCG on the risk of severe COVID-19. Prospective clinical trials are necessary to demonstrate whether BCG may have an immediate protective effect. Despite the attractiveness of the concept, the proposal of BCG trials for protection against COVID-19 has also raised the concern that the strengthened cytokine response may lead to deleterious effects in some patients. The major clinical complication in patients with COVID-19 is respiratory insufficiency due to local hyperinflammation and acute respiratory distress syndrome (ARDS).[37] Indeed, recent studies argue for an increased systemic inflammatory reaction in patients with severe SARS-CoV-2 infection: circulating concentrations of proinflammatory cytokines such as IL-6, TNF-α, MCP1, MIP1α, and IP10 are increased in COVID-19 patients on the intensive care unit (ICU) when compared to those who do not need ICU admission.[1] This strong increase in systemic inflammation is associated with endothelial dysfunction as assessed by D-dimers,[37] and hyperactive CCR6$^+$Th17$^+$ T cells locally in the lung.[38] Administration of anti-IL-6R antibodies[39,40] or IL-1R antagonist (anakinra)[41,42] in patients with COVID-19 and hyperinflammation seems to have beneficial effects. These observations

Figure 4. Association between BCG-Induced Immune Memory and Self-Reported Sickness and Symptoms during the SARS-CoV-2 Pandemic

(A) Inter-individual variability of immune memory responses. Trained immunity was assessed by the fold change in *ex vivo S. aureus*-induced TNF-α (n = 195) and *S. aureus*- and *M. tuberculosis* (*Mtb*)-induced IL-6 (n = 195 and n = 193, respectively) cytokine production 3 months after BCG vaccination as compared to levels before vaccination. The adaptive immune memory response was assessed by the fold change in *Mtb*-induced IFN-γ production (n = 189). Fold changes in cytokine production are shown in boxplots, red line indicates the non-responder/responder cut off (1.2 for trained immunity, 1.5 for adaptive immunity). Boxes represent middle 50% of the data with a center line for the median, the lines extending the boxes roughly represent the 95% confidence interval for comparing medians, outliers are shown as separate dots.

(B–D) Individuals with a fold change of 1.2 or higher were considered responders, individuals with a fold change smaller than 1.2 were considered non-responders. Proportion of non-responders and responders that reported sickness (B; *S. aureus*-induced IL-6, non-responder, n = 128; responder, n = 67, *Mtb*-induced IL-6, non-responder, n = 112; responder, n = 70), *S. aureus*-induced TNF-α, non-responder, n = 94; responder, n = 101) or any symptoms (C; *S. aureus*-induced IL-6, non-responder, n = 128; responder, n = 67), *Mtb*-induced IL-6, non-responder, n = 112; responder, n = 70) are shown (p = chi-square test p value). (D) Percentage of *S. aureus*-induced IL-6 cytokine fold change non-responders and responders that reported a specific symptom (non-responder, n = 128; responder, n = 67; p = Fisher's exact test p value).

(E–G) Percentage of *Mtb*-induced IFN-γ cytokine fold change non-responders and responders that reported sickness (E) or symptoms (F) (non-responder, n = 93; responder, n = 96; p = chi-square test p value). (G) Percentage of *Mtb*-induced IFN-γ cytokine fold change non-responders and responders that reported a specific symptom (non-responder, n = 93; responder, n = 96; p = Fisher's exact test p value).

See also Figures S5 and S6.

CellPress
OPEN ACCESS

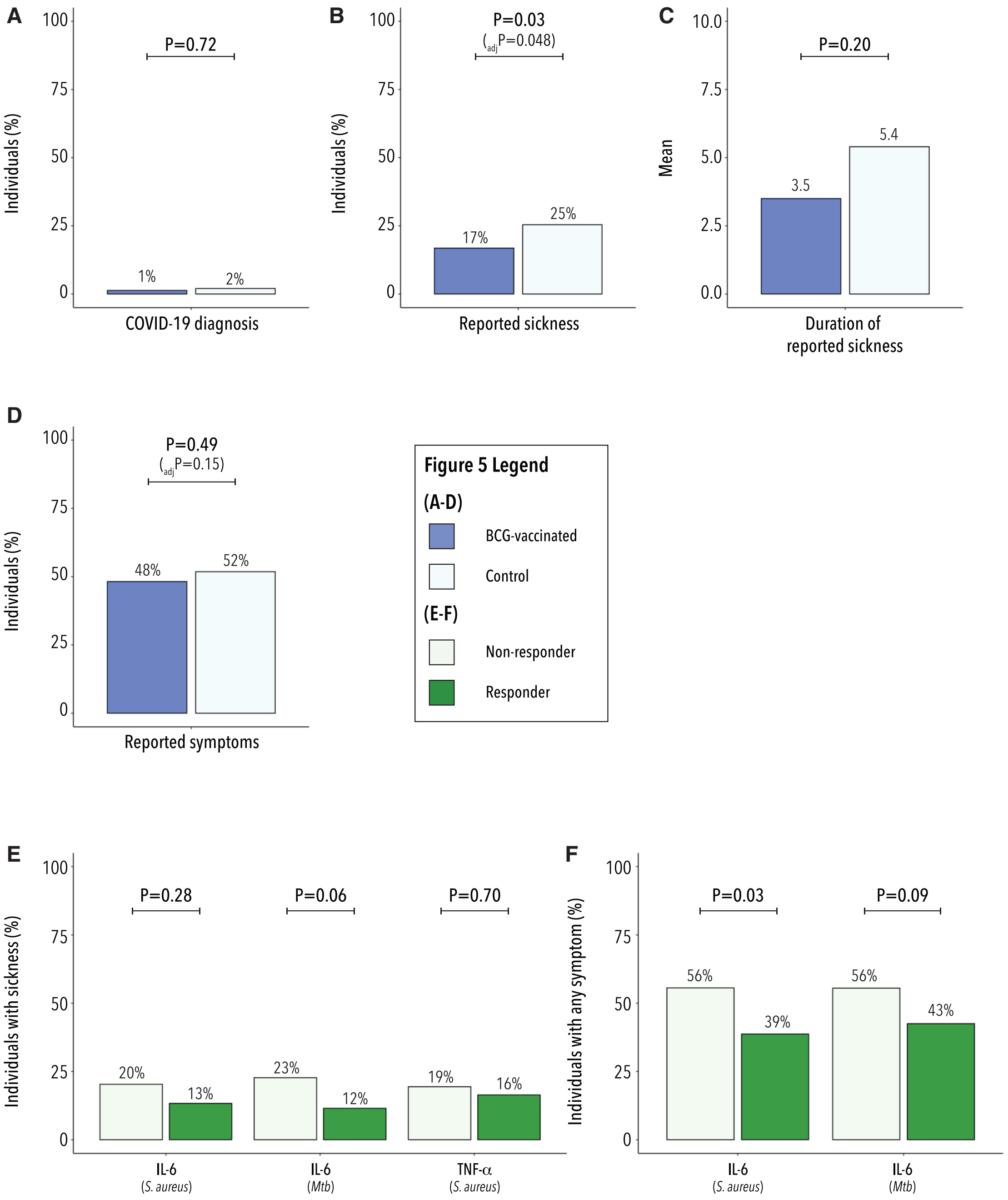

Figure 5. Summary of Main Results Analyzed for the First 4 Weeks of the COVID-19 Outbreak in the Netherlands, in which More Individuals Responded to the Survey

(A) Percentage of participants with positive COVID-19 diagnosis in the BCG-vaccinated (n = 309) and control group (n = 197) (p = Fisher's exact test p value).
(B) Percentage of self-reported sickness between February 27th and March 31st 2020 in the BCG-vaccinated (n = 309) and control group (n = 197) (p = chi-square test p value; $_{adj}$p = logistic regression adjusted p value).
(C) The average number of reported days of sickness in the BCG-vaccinated and control group (n = 102; p = Wilcoxon rank-sum test p value).
(D) Percentage of individuals that reported any symptom in the BCG-vaccinated and control group (n = 506; p = chi-square test p value).
(E and F) Trained immunity was assessed by the fold change in *ex vivo S. aureus*-induced TNF-α (n = 208) and *S. aureus*- and *M. tuberculosis* (*Mtb*)-induced IL-6 (n = 208 and n = 206, respectively) cytokine production 3 months after BCG vaccination as compared to levels before vaccination. Individuals with a fold change of 1.2 or higher were considered responders, individuals with a fold change smaller than 1.2 were considered non-responders. Percentage of non-responders (*S. aureus*-induced TNF-α, n = 98; *S. aureus*-induced IL-6, n = 133; *Mtb*-induced IL-6, n = 119) and responders (*S. aureus*-induced TNF-α, n = 110; *S. aureus*-induced IL-6, n = 75; *Mtb*-induced IL-6, n = 87) that reported sickness (E), or symptoms (F) are shown (p = chi-square test p value).

necessitate a careful assessment of the safety of BCG vaccination in the context of COVID-19 infection, before very large clinical trials are initiated.

We approached the important aspect of the safety profile of BCG vaccination during the COVID-19 pandemic by comparing the incidence and severity of COVID-19 symptoms in individuals either vaccinated with BCG in the last 5 years or not. BCG vaccination was not associated with any increase in incidence of disease or severity of symptoms during the COVID-19 outbreak. Serological data in the Netherlands indicated that between 5.5%–10.0% of the population has been infected. Moreover, Nijmegen is situated very close to the epicenter of the pandemic in the Netherlands, and thus these percentages are likely to be higher for our study population. This means that at least 14–27, and likely more, individuals in the BCG group have been infected during the first pandemic wave. The fact that none of these individuals have needed hospitalization is a strong argument for the safety of BCG vaccination. If anything, our data suggest that BCG vaccination might be associated with a decrease in the incidence of sickness during the COVID-19 pandemic, as well as lower incidence of severe fatigue. The significant reduction in sickness by BCG vaccination is in line with the earlier studies in children[5,8] and in adults[10] that reported a decrease in respiratory tract infections.

The lack of deleterious effects of BCG vaccination is not completely unexpected. In healthy individuals who are vaccinated with BCG, and in which the innate antimicrobial mechanisms would be boosted by trained immunity, this likely leads to decreased viremia, faster viral elimination, and subsequently decreased inflammation, fewer symptoms, and quicker recovery. This chain of events was, indeed, also recorded in a model of experimental infection with the yellow fever vaccine, in which BCG vaccination decreased viral loads and systemic inflammation.[12] In contrast, an initial defective antiviral response in some individuals at risk (e.g., the elderly) can lead to high viremia, deleterious systemic inflammation, and severe disease. It is reassuring to observe that these theoretical considerations are supported by actual clinical data.

In conclusion, in the present study, we demonstrate that BCG vaccination is safe and does not lead to increased morbidity due to COVID-19. Prospective randomized clinical trials to study the impact of BCG vaccination on morbidity and mortality due to SARS-CoV-2 infection are urgently needed before BCG vaccination can be recommended against COVID-19.[13] Importantly, two indispensable additional pieces in the puzzle of BCG non-specific effects are provided by complementary studies by our group: one study demonstrates the induction of trained immunity by BCG revaccination in older adults from a sub-Saharan country (Berendsen et al. and M.G.N., unpublished data), while a randomized clinical trial in Greece, in an elderly population, shows significantly lower incidence of any infection, with 80% reduction in respiratory tract infections (E.J.G.-B. et al. and M.G.N. unpublished data). Based on all these studies, one could envisage induction of trained immunity as an important tool against emerging dangerous pathogens: BCG (or other stimuli that induce trained immunity) could be rapidly tested and eventually employed in the beginning of a pandemic, bridging the 1- to 2-year period needed for the development of a pathogen-specific vaccine. Indeed, a specific vaccine is needed and will hopefully provide a long-term solution for COVID-19, because protection through induction of trained immunity by BCG is likely to be relatively short (for 2–3 years), as also suggested by a recent epidemiological study.[43] This concept carries particular force at this time, because there is an urgent need to develop strategies to restrain SARS-CoV2 and limit the pandemic, which has put half of the Earth's population under quarantine.

Limitations of Study

Caution is warranted in interpreting our findings: limitations include the retrospective nature of the study in two relatively small groups of volunteers, and the potential for selection bias. Furthermore, a microbiological diagnosis was absent in the majority of the individuals, and we may have captured sickness and symptoms unrelated to COVID-19. For these reasons, the data in this study indicate that BCG vaccination is safe during the COVID-19 outbreak, but does not permit conclusions regarding a potential beneficial effect of BCG against SARS-CoV-2 infection. Whether BCG has a protective effect specifically against COVID-19 needs to be investigated in future randomized clinical trials.

STAR★METHODS

Detailed methods are provided in the online version of this paper and include the following:

- KEY RESOURCES TABLE
- RESOURCE AVAILABILITY
  - Lead Contact
  - Materials Availability
- EXPERIMENTAL MODEL AND SUBJECT DETAILS
  - Human Subjects
- METHOD DETAILS
  - Peripheral blood mononuclear cell isolation and stimulation
  - Protein measurements
- QUANTIFICATION AND STATISTICAL ANALYSIS
  - Logistic regression
  - Statistics cytokine production and circulating mediators

SUPPLEMENTAL INFORMATION

Supplemental Information can be found online at https://doi.org/10.1016/j.xcrm.2020.100073.

ACKNOWLEDGMENTS

M.G.N. was supported by an ERC Advanced Grant (833247) and a Spinoza Grant of the Netherlands Association for Scientific Research.

AUTHOR CONTRIBUTIONS

Methodology, S.J.C.F.M.M., R.C.v.D., M.J., P.D., E.T., V.P.M., V.A.C.M.K., L.C.J.d.B., M.C.C., S.S., and M.O.; Investigation, R.C.v.D. and S.J.C.F.M.M.; Writing – Original Draft, M.G.N. and S.J.C.F.M.M.; Writing – Review & Editing, R.C.v.D., C.H.v.W., T.v.D., F.L.v.d.V., L.A.B.J., J.t.O., J.W.M.v.d.M., N.C., P.A., C.S.-B., E.J.G.-B., M.B., and R.v.C.; Supervision, M.G.N.

DECLARATION OF INTERESTS

M.G.N. and L.A.B.J. are scientific founders of TTxD.

Received: May 15, 2020
Revised: June 12, 2020
Accepted: July 26, 2020
Published: August 5, 2020

REFERENCES

1. Huang, C., Wang, Y., Li, X., Ren, L., Zhao, J., Hu, Y., Zhang, L., Fan, G., Xu, J., Gu, X., et al. (2020). Clinical features of patients infected with 2019 novel coronavirus in Wuhan, China. Lancet *395*, 497–506.
2. World Health Organization (2020). WHO Timeline-COVID-19. https://www.who.int/news-room/detail/27-04-2020-who-timeline—covid-19.
3. World Health Organization (2020). Coronavirus disease (COVID-19): situation report-103. https://www.who.int/docs/default-source/coronaviruse/situation-reports/20200502-covid-19-sitrep-103.pdf?sfvrsn=d95e76d8_6.
4. Netea, M.G., Joosten, L.A., Latz, E., Mills, K.H., Natoli, G., Stunnenberg, H.G., O'Neill, L.A., and Xavier, R.J. (2016). Trained immunity: A program of innate immune memory in health and disease. Science *352*, aaf1098.
5. Aaby, P., Roth, A., Ravn, H., Napirna, B.M., Rodrigues, A., Lisse, I.M., Stensballe, L., Diness, B.R., Lausch, K.R., Lund, N., et al. (2011). Randomized trial of BCG vaccination at birth to low-birth-weight children: beneficial nonspecific effects in the neonatal period? J. Infect. Dis. *204*, 245–252.
6. Biering-Sørensen, S., Aaby, P., Napirna, B.M., Roth, A., Ravn, H., Rodrigues, A., Whittle, H., and Benn, C.S. (2012). Small randomized trial among low-birth-weight children receiving bacillus Calmette-Guérin vaccination at first health center contact. Pediatr. Infect. Dis. J. *31*, 306–308.
7. Higgins, J.P., Soares-Weiser, K., López-López, J.A., Kakourou, A., Chaplin, K., Christensen, H., Martin, N.K., Sterne, J.A., and Reingold, A.L. (2016). Association of BCG, DTP, and measles containing vaccines with childhood mortality: systematic review. BMJ *355*, i5170.
8. Biering-Sørensen, S., Aaby, P., Lund, N., Monteiro, I., Jensen, K.J., Eriksen, H.B., Schaltz-Buchholzer, F., Jørgensen, A.S.P., Rodrigues, A., Fisker, A.B., and Benn, C.S. (2017). Early BCG-Denmark and Neonatal Mortality Among Infants Weighing <2500 g: A Randomized Controlled Trial. Clin. Infect. Dis. *65*, 1183–1190.
9. Stensballe, L.G., Nante, E., Jensen, I.P., Kofoed, P.E., Poulsen, A., Jensen, H., Newport, M., Marchant, A., and Aaby, P. (2005). Acute lower respiratory tract infections and respiratory syncytial virus in infants in Guinea-Bissau: a beneficial effect of BCG vaccination for girls community based case-control study. Vaccine *23*, 1251–1257.
10. Wardhana, D.E.A., Datau, E.A., Sultana, A., Mandang, V.V., and Jim, E. (2011). The efficacy of Bacillus Calmette-Guerin vaccinations for the prevention of acute upper respiratory tract infection in the elderly. Acta Med. Indones. *43*, 185–190.
11. Ohrui, T., Nakayama, K., Fukushima, T., Chiba, H., and Sasaki, H. (2005). [Prevention of elderly pneumonia by pneumococcal, influenza and BCG vaccinations]. Nippon Ronen Igakkai Zasshi *42*, 34–36.
12. Arts, R.J.W., Moorlag, S., Novakovic, B., Li, Y., Wang, S.Y., Oosting, M., Kumar, V., Xavier, R.J., Wijmenga, C., Joosten, L.A.B., et al. (2018). BCG Vaccination Protects against Experimental Viral Infection in Humans through the Induction of Cytokines Associated with Trained Immunity. Cell Host Microbe *23*, 89–100.
13. Curtis, N., Sparrow, A., Ghebreyesus, T.A., and Netea, M.G. (2020). Considering BCG vaccination to reduce the impact of COVID-19. Lancet *395*, 1545–1546.
14. Ye, Q., Wang, B., and Mao, J. (2020). The pathogenesis and treatment of the 'Cytokine Storm' in COVID-19. J. Infect. *80*, 607–613.
15. Chang, D., Xu, H., Rebaza, A., Sharma, L., and Dela Cruz, C.S. (2020). Protecting health-care workers from subclinical coronavirus infection. Lancet Respir. Med. *8*, e13.
16. Chou, R., Dana, T., Buckley, D.I., Selph, S., Fu, R., and Totten, A.M. (2020). Epidemiology of and Risk Factors for Coronavirus Infection in Health Care Workers: A Living Rapid Review. Ann. Intern. Med. *173*, 120–136.
17. RIVM (2020). Current information about COVID-19 (novel coronavirus). https://www.rivm.nl.
18. RIVM (2020). Epidemiologische situatie COVID-19 Nederland 08-06-2020. https://www.rivm.nl/sites/default/files/2020-06/COVID-19_WebSite_rapport_dagelijks20200608_1021.pdf.
19. Wikman, A., Marklund, S., and Alexanderson, K. (2005). Illness, disease, and sickness absence: an empirical test of differences between concepts of ill health. J. Epidemiol. Community Health *59*, 450–454.
20. Wu, Z., and McGoogan, J.M. (2020). Characteristics of and Important Lessons From the Coronavirus Disease 2019 (COVID-19) Outbreak in China: Summary of a Report of 72314 Cases From the Chinese Center for Disease Control and Prevention. JAMA, Published online February 24, 2020. https://doi.org/10.1001/jama.2020.2648.
21. Moorlag, S., Arts, R.J.W., van Crevel, R., and Netea, M.G. (2019). Non-specific effects of BCG vaccine on viral infections. Clin. Microbiol. Infect. *25*, 1473–1478.
22. Kleinnijenhuis, J., Quintin, J., Preijers, F., Joosten, L.A., Ifrim, D.C., Saeed, S., Jacobs, C., van Loenhout, J., de Jong, D., Stunnenberg, H.G., et al. (2012). Bacille Calmette-Guerin induces NOD2-dependent nonspecific protection from reinfection via epigenetic reprogramming of monocytes. Proc. Natl. Acad. Sci. USA *109*, 17537–17542.
23. Arts, R.J.W., Carvalho, A., La Rocca, C., Palma, C., Rodrigues, F., Silvestre, R., Kleinnijenhuis, J., Lachmandas, E., Gonçalves, L.G., Belinha, A., et al. (2016). Immunometabolic Pathways in BCG-Induced Trained Immunity. Cell Rep. *17*, 2562–2571.
24. Buffen, K., Oosting, M., Quintin, J., Ng, A., Kleinnijenhuis, J., Kumar, V., van de Vosse, E., Wijmenga, C., van Crevel, R., Oosterwijk, E., et al. (2014). Autophagy controls BCG-induced trained immunity and the response to intravesical BCG therapy for bladder cancer. PLoS Pathog. *10*, e1004485.
25. Green, C.M., Fanucchi, S., Fok, E.T., Moorlag, S.J.C.F.M., Dominguez-Andres, J., Negishi, Y., Joosten, L.A.B., Netea, M.G., and Mhlanga, M.M. (2020). COVID-19: A model correlating BCG vaccination to protection from mortality implicates trained immunity. medRxiv. https://doi.org/10.1101/2020.04.10.20060905.
26. Netea, M.G., Giamarellos-Bourboulis, E.J., Domínguez-Andrés, J., Curtis, N., van Crevel, R., van de Veerdonk, F.L., and Bonten, M. (2020). Trained Immunity: a Tool for Reducing Susceptibility to and the Severity of SARS-CoV-2 Infection. Cell *181*, 969–977.
27. O'Neill, L.A.J., and Netea, M.G. (2020). BCG-induced trained immunity: can it offer protection against COVID-19? Nat. Rev. Immunol. *20*, 335–337.
28. Spencer, J.C., Ganguly, R., and Waldman, R.H. (1977). Nonspecific protection of mice against influenza virus infection by local or systemic immunization with Bacille Calmette-Guérin. J. Infect. Dis. *136*, 171–175.
29. Starr, S.E., Visintine, A.M., Tomeh, M.O., and Nahmias, A.J. (1976). Effects of immunostimulants on resistance of newborn mice to herpes simplex type 2 infection. Proc. Soc. Exp. Biol. Med. *152*, 57–60.
30. Cirovic, B., de Bree, L.C.J., Groh, L., Blok, B.A., Chan, J., van der Velden, W.J.F.M., Bremmers, M.E.J., van Crevel, R., Händler, K., Picelli, S., et al. (2020). BCG Vaccination in Humans Elicits Trained Immunity via the Hematopoietic Progenitor Compartment. Cell Host Microbe, Published online June 9, 2020. https://doi.org/10.1016/j.chom.2020.05.014.
31. Joosten, S.A., van Meijgaarden, K.E., Arend, S.M., Prins, C., Oftung, F., Korsvold, G.E., Kik, S.V., Arts, R.J., van Crevel, R., Netea, M.G., and Ottenhoff, T.H. (2018). Mycobacterial growth inhibition is associated with trained innate immunity. J. Clin. Invest. *128*, 1837–1851.

32. Netea, M.G., Domínguez-Andrés, J., Barreiro, L.B., Chavakis, T., Divangahi, M., Fuchs, E., Joosten, L.A.B., van der Meer, J.W.M., Mhlanga, M.M., Mulder, W.J.M., et al. (2020). Defining trained immunity and its role in health and disease. Nat. Rev. Immunol. *20*, 375–388.

33. Miller, A., Reandelar, M.J., Fasciglione, K., Roumenova, V., Li, Y., and Otazu, G.H. (2020). Correlation between universal BCG vaccination policy and reduced morbidity and mortality for COVID-19: an epidemiological study. medRxiv. https://doi.org/10.1101/2020.03.24.20042937.

34. Shet, A., Ray, D., Malavige, N., Santosham, M., and Bar-Zeev, N. (2020). Differential COVID-19-attributable mortality and BCG vaccine use in countries. medRxiv. https://doi.org/10.1101/2020.04.01.20049478.

35. Sala, G., Chakraborti, R., Ota, A., and Miyakawa, T. (2020). Association of BCG vaccination policy with prevalence and mortality of COVID-19. medRxiv. https://doi.org/10.1101/2020.03.30.20048165.

36. Faust, L.H.S., MacLean, E., and Svadzian, A. (2020). Universal BCG vaccination and protection against COVID-19: critique of an ecological study. https://naturemicrobiologycommunity.nature.com/posts/64892-universal-bcg-vaccination-and-protection-against-covid-19-critique-of-an-ecological-study.

37. Zhou, F., Yu, T., Du, R., Fan, G., Liu, Y., Liu, Z., Xiang, J., Wang, Y., Song, B., Gu, X., et al. (2020). Clinical course and risk factors for mortality of adult inpatients with COVID-19 in Wuhan, China: a retrospective cohort study. Lancet *395*, 1054–1062.

38. Xu, Z., Shi, L., Wang, Y., Zhang, J., Huang, L., Zhang, C., Liu, S., Zhao, P., Liu, H., Zhu, L., et al. (2020). Pathological findings of COVID-19 associated with acute respiratory distress syndrome. Lancet Respir. Med. *8*, 420–422.

39. Xu, X., Han, M., Li, T., Sun, W., Wang, D., Fu, B., Zhou, Y., Zheng, X., Yang, Y., Li, X., et al. (2020). Effective treatment of severe COVID-19 patients with tocilizumab. Proc. Natl. Acad. Sci. USA *117*, 10970–10975.

40. Sciascia, S., Aprà, F., Baffa, A., Baldovino, S., Boaro, D., Boero, R., Bonora, S., Calcagno, A., Cecchi, I., Cinnirella, G., et al. (2020). Pilot prospective open, single-arm multicentre study on off-label use of tocilizumab in patients with severe COVID-19. Clin. Exp. Rheumatol. *38*, 529–532.

41. Cavalli, G., De Luca, G., Campochiaro, C., Della-Torre, E., Ripa, M., Canetti, D., Oltolini, C., Castiglioni, B., Tassan Din, C., Boffini, N., et al. (2020). Interleukin-1 blockade with high-dose anakinra in patients with COVID-19, acute respiratory distress syndrome, and hyperinflammation: a retrospective cohort study. Lancet Rheumatol *2*, e325–e331.

42. Giamarellos-Bourboulis, E.J., Netea, M.G., Rovina, N., Akinosoglou, K., Antoniadou, A., Antonakos, N., Damoraki, G., Gkavogianni, T., Adami, M.E., Katsaounou, P., et al. (2020). Complex Immune Dysregulation in COVID-19 Patients with Severe Respiratory Failure. Cell Host Microbe *27*, 992–1000.

43. Hamiel, U., Kozer, E., and Youngster, I. (2020). SARS-CoV-2 Rates in BCG-Vaccinated and Unvaccinated Young Adults. JAMA *323*, 2340–2341.

44. Assarsson, E., Lundberg, M., Holmquist, G., Björkesten, J., Thorsen, S.B., Ekman, D., Eriksson, A., Rennel Dickens, E., Ohlsson, S., Edfeldt, G., et al. (2014). Homogenous 96-plex PEA immunoassay exhibiting high sensitivity, specificity, and excellent scalability. PLoS ONE *9*, e95192.

45. Li, Y., Oosting, M., Smeekens, S.P., Jaeger, M., Aguirre-Gamboa, R., Le, K.T.T., Deelen, P., Ricano-Ponce, I., Schoffelen, T., Jansen, A.F.M., et al. (2016). A Functional Genomics Approach to Understand Variation in Cytokine Production in Humans. Cell *167*, 1099–1100.

STAR★METHODS

KEY RESOURCES TABLE

REAGENT or RESOURCE	SOURCE	IDENTIFIER
Bacterial and Virus Strains		
Heat-killed *Mycobacterium tuberculosis*	Gift	H37Rv
Bacille Calmette-Guérin Vaccine	Intervax	Bulgaria strain
Bacille Calmette-Guérin Vaccine	SSI	1331
Staphylococcus aureus	Gift	Clinical isolate
Chemicals, Peptides, and Recombinant Proteins		
Ficoll-Paque	GE Healthcare	Cat#17-1440-03
Roswell Park Memorial Institute medium (RPMI)	Invitrogen	Cat#22406031
Critical Commercial Assays		
Human TNFα ELISA	R&D systems	Cat#DY210
Human IL-6 ELISA	Sanquin	Cat#M1916
Human IFNγ ELISA	Sanquin	Cat#M1933
Software and Algorithms		
R version 3.6.1	R Foundation for Statistical Computing	https://www.R-project.org/
Castor	Castor EDC	https://www.castoredc.com

RESOURCE AVAILABILITY

Lead Contact

Further information and requests for resources and reagents should be directed to and will be fulfilled by the Lead Contact, Mihai G. Netea at the Radboud University Medical Center, Nijmegen, the Netherlands (mihai.netea@radboudumc.nl).

Materials Availability

This study did not generate new unique reagents.

EXPERIMENTAL MODEL AND SUBJECT DETAILS

Human Subjects

This retrospective cohort study was performed in cohorts of healthy adults (both females and males) of Western European descent that are part of the Human Functional Genomics Project (300BCG cohort, no. NL58553.091.16, 500FG cohort, no. NL42561.091.12, BCG booster cohort, no. NL58219.091.16). The study was approved by the Ethical Committee (CMO) of Radboud University Nijmegen, the Netherlands. Inclusion of volunteers and experiments were conducted according to the principles expressed in the Declaration of Helsinki. All volunteers gave written informed consent before any material was taken.

The 500FG cohort consists of 534 healthy individuals (237 males and 296 females) that were included in the period between 8/2013 and 12/2014. 511 participants gave permission upon enrollment in the 500FG study to be approached for future research studies. Ages range from 18 to 75, the majority of the individuals were 30 years or younger (n = 421). The 300BCG cohort consists of 326 healthy individuals (141 males and 185 females) that were included between 4/2017 and 6/2018. The age range of this cohort ranges from 18 to 71 with the majority of individuals (n = 279) being younger than 30 years of age. The BCG booster cohort contained 31 healthy individuals (age range 19 – 53 year.) that were included between 12/2019 and 2/2020. In all cohorts, participants were included at the Radboud University Medical Center (Radboudumc), the Netherlands. Recruitment of participants was performed in a similar way for all cohorts: via flyers and posters at the Radboudumc and the Radboud University.

29 individuals that participated in the 500FG study also participated later in the 300BCG study and received a BCG vaccination. These participants were therefore considered 300BCG participants in this study. As participants from the 500FG cohort were included approximately 7 years ago, a lower response rate might be expected compared to the more recent 300BCG and BCG booster cohorts. Indeed, for 22 individuals from the 500FG cohort the e-mail domain no longer existed and for this reason they did not receive the survey invitation. As not all domains allow for such checks it is plausible that this number is underestimated (e.g., due to change in e-mail address). Participants of the 300BCG and BCG booster cohorts were included more recently (2017-2020).

A digital survey was sent to all participants of the 500FG (n = 460), 300BCG (n = 326) and BCG booster (n = 31) cohorts on April 1st 2020 and the possibility to fill in the survey closed on April 14th 2020. The response rate of the 500FG cohort was 53.7% (n = 247) and the response rate of the 300BCG and BCG booster cohorts was 79.8% (n = 260) and 61.3% (n = 19) respectively for the first survey. The second survey was sent only to those individuals that completed the first survey. 241 individuals from the 300BCG cohort, 207 individuals from the 500FG cohort and 19 individuals from the BCG booster cohort completed both surveys.

In total, 197 individuals had not received the BCG vaccine (1 from the 300BCG cohort, 192 from the 500FG cohort and 4 from the BCG booster cohort), 329 individuals had received BCG (259 from the 300BCG cohort, 55 from the 500FG cohort and 15 from the BCG booster cohort). In order to assess the effect of recent BCG vaccination, 20 individuals (all part of the 500FG cohort) that received the BCG vaccine before 2015 were excluded from all analyses. The resulting dataset containing response to the first survey consisted of 309 BCG-vaccinated individuals and 197 controls.

Participants completed a digital survey in which they were queried about sickness and symptoms between February 27th and March 31st 2020. This period was chosen as the first COVID-19 positive case was detected in the Netherlands on February 27th 2020 and the peak of people admitted to the hospital due to COVID-19 was at the end of March 2020. In addition, recent travel history and previous contact with SARS-CoV-2 infected individuals were questioned to ascertain possible exposure differences. Finally, individuals were asked if they had received a BCG vaccination and if so, when they were vaccinated. Participants that responded to the first digital survey all received a second survey in which they were queried about COVID-19 related disease, sickness and symptoms in the period 1st April – 30th of April. Data depicted in Figures 1, 2, 3, and 4 contain the results from participants that completed both surveys (BCG-vaccinated n = 266; control n = 164). Figure 5 shows the data based on the results of survey 1 and represent the time period between February 27th and March 31st. Participants from the BCG booster cohort received a second BCG vaccination in April and were for this reason excluded from analysis when the time period February 27th – April 30th was assessed (Figures 1, 2, 3, and 4).

METHOD DETAILS

Peripheral blood mononuclear cell isolation and stimulation

Peripheral blood mononuclear cells (PBMCs) were isolated from EDTA whole blood with Ficoll-Paque (GE healthcare, UK) density gradient separation. Cells were washed twice in phosphate buffered saline (PBS) and counted with a Sysmex hematology analyzer (XN-450). PBMCs were suspended in Dutch modified RPMI 1640 medium (Roswell Park Memorial Institute, Invitrogen, CA, USA), supplemented with 50 μg/mL gentamicin, 2 mM Glutamax (GIBCO) and 1 mM pyruvate (GIBCO). 5×10^5 PBMCs were added to round bottom 96-well plates (Greiner) in a final volume of 200 μL/well and incubated either with culture medium only as a negative control or heat-killed *Mycobacterium tuberculosis* (*M. tuberculosis* or *Mtb*) H37Rv (5 μg/mL, specific stimulus), or heat-killed *Staphylococcus aureus* (*S. aureus*) (10^6 CFU/mL, non-specific stimulus). After 24 hours and 7 days of incubation at 37°C, supernatants were collected and stored at −20°C until analysis. Cytokine production was measured in 24 hours (IL-6, and TNF-α) and 7 days (IFN-γ) supernatants using commercial ELISA kits in accordance with the manufacturer's instructions. In the 300BCG cohort, PBMCs were isolated and stimulated before vaccination and 3 months after BCG vaccination.

Protein measurements

Circulating plasma inflammatory markers were assessed using the commercially available Olink Proteomics AB (Uppsala Sweden), using a Proseek © Multiplex proximity extension assay.[44] Detected proteins are normalized and measured on a log2-scale as normalized protein expression values.

QUANTIFICATION AND STATISTICAL ANALYSIS

Questionnaire data was exported from Castor and analyzed in R version 3.6.1, using dplyr, tidyr, tidyverse and reshape2 for data inspection and transformation, ggplot2 and ggpubr for visualizations including statistics and rcompanion for more in-depth statistical analyses. Chi-square tests were used for comparisons of categorical demographic characteristics (Table 1) (e.g., sex, age, BMI). Finally, differences in distribution of BCG-vaccinated individuals and controls and various dependent variables (Figures 2 and 4) were assessed by either Chi-square tests or, when the expected count in one of the cells of the 2x2 table was less than 5 for at least one of the symptoms, by Fisher's Exact tests. Statistical test and n are specified in each figure legend, asterisks indicate statistical significance (*, $p < 0.05$; **, $p < 0.01$; ***, $p < 0.001$).

Logistic regression

For the comparison of reported sickness and symptoms between the groups, we created a logistic regression model using the glm-formula with family = binomial for the dependent variable reported sickness with predictors BCG vaccination status, age, presence of underlying chronic condition (none versus one or more), healthcare-work (yes versus no), known contact with corona-infected person (yes versus no) and any international travel between January 1st and March 31st 2020 (yes versus no). The fit of the model was assessed by means of a givitCalibrationBelt-plot using the package givitiR (see Figure S3).

Statistics cytokine production and circulating mediators

A multitude of evidence shows that both age and sex can influence circulating cytokines.[45] In order to properly compare circulating mediator (log2(NPX)) values, that had been assessed separately for the two cohorts and as such were normalized within cohort, we created a linear model for the log-transformed values, using cohort, age and sex as predictors. The resulting residual circulating mediators were visualized in grouped boxplots both for reported sickness and any symptoms in the period between February 27th and April 30th 2020. Wilcoxon-rank sum tests were performed to test for differences in mean cytokine production upon stimulation and differences in mean circulating mediator.

The magnitude of the trained immunity (innate immune memory) response was assessed by the fold change in cytokine production (TNF-α, IL-6) three months after BCG vaccination as compared to levels before vaccination. The specific immune response (adaptive immune memory) was assessed by the fold change in IFN-γ production upon stimulation with *M. tuberculosis*. The fold change for each cytokine-stimulus combination was dichotomized based on a threshold of 1.2 for IL-6 and TNF-α, and 1.5 for IFN-γ, where individuals below the threshold were categorized as non-responders and individuals above the threshold as responders. We tested for distribution differences of responders in reported disease and any symptoms in the indicated time period by means of Chi-square tests or Fisher's Exact test when sample size was too small.

Cell Reports Medicine, Volume *1*

Supplemental Information

Safety and COVID-19 Symptoms in Individuals Recently Vaccinated with BCG: a Retrospective Cohort Study

Simone J.C.F.M. Moorlag, Rosanne C. van Deuren, Cornelis H. van Werkhoven, Martin Jaeger, Priya Debisarun, Esther Taks, Vera P. Mourits, Valerie A.C.M. Koeken, L. Charlotte J. de Bree, Thijs ten Doesschate, Maartje C. Cleophas, Sanne Smeekens, Marije Oosting, Frank L. van de Veerdonk, Leo A.B. Joosten, Jaap ten Oever, Jos W.M. van der Meer, Nigel Curtis, Peter Aaby, Christine Stabell-Benn, Evangelos J. Giamarellos-Bourboulis, Marc Bonten, Reinout van Crevel, and Mihai G. Netea

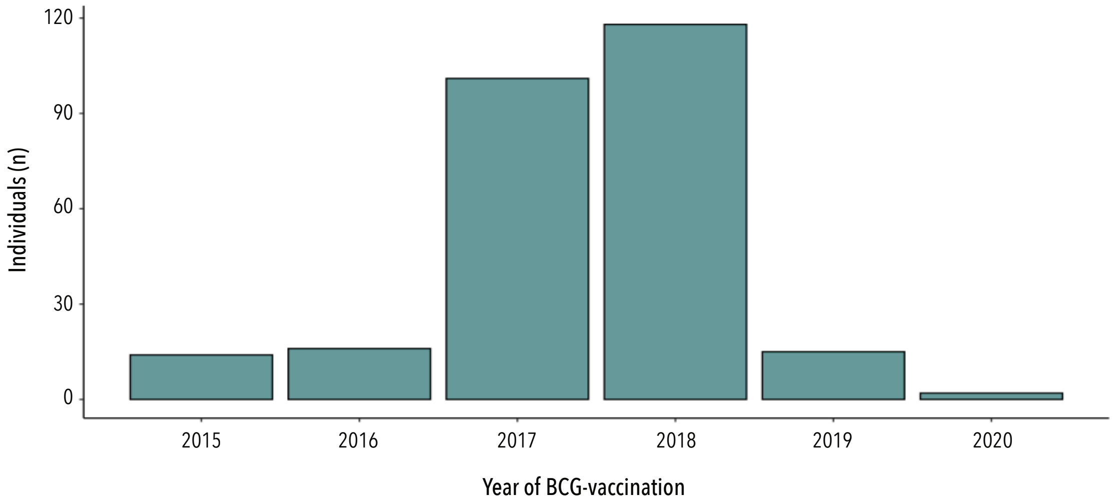

Figure S1 - Timing of BCG vaccination (n=266). Individuals vaccinated in 2017 and 2018 were vaccinated with BCG Intervax (Canada), in all other years individuals were vaccinated with BCG SSI (Denmark). Related to Table 1.

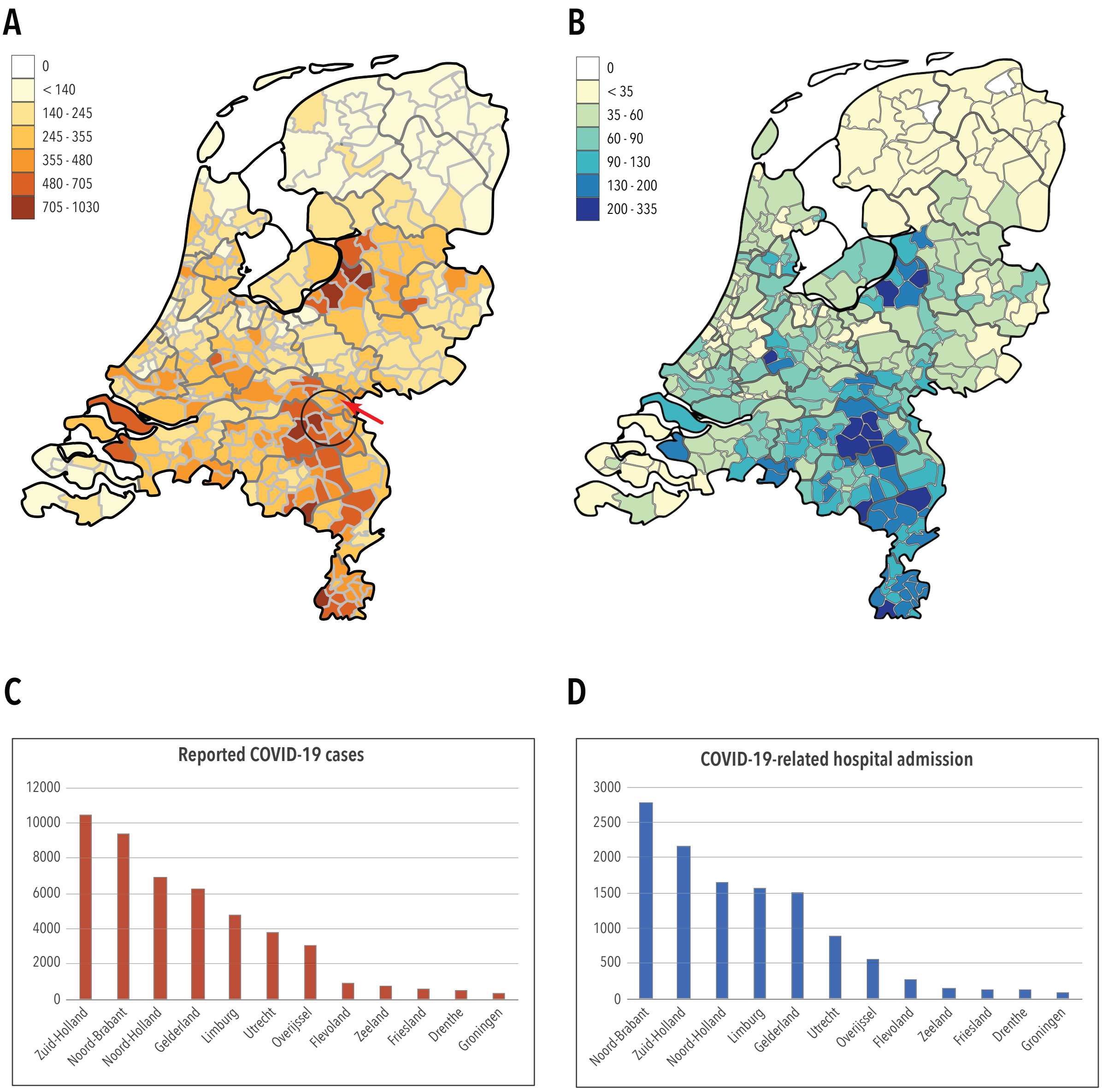

Figure S2 - Distribution of reported COVID-19 cases and COVID-19-related hospital admission in the Netherlands. (A) Reported COVID-19 cases in the Netherlands per 100000 inhabitants, as of June 8, 2020. The actual number of COVID-19 cases is higher as not all individuals are tested. Participants from all cohorts in this study were included at the Radboud University Medical Center (Radboudumc) in Nijmegen (red arrow). Most study participants live in the area indicated by the black circle. (B) Reported number of COVID-19 patients admitted to the hospital per 100000 inhabitants. (C) COVID-19 reported cases in the Netherlands as of June 8, 2020, by province. (D) COVID-19-related hospital admission in the Netherlands as of June 8, 2020, by province. Related to Figure 2.
Source: RIVM: Epidemiologische situatie COVID-19. Nederland. 08-06-2020.

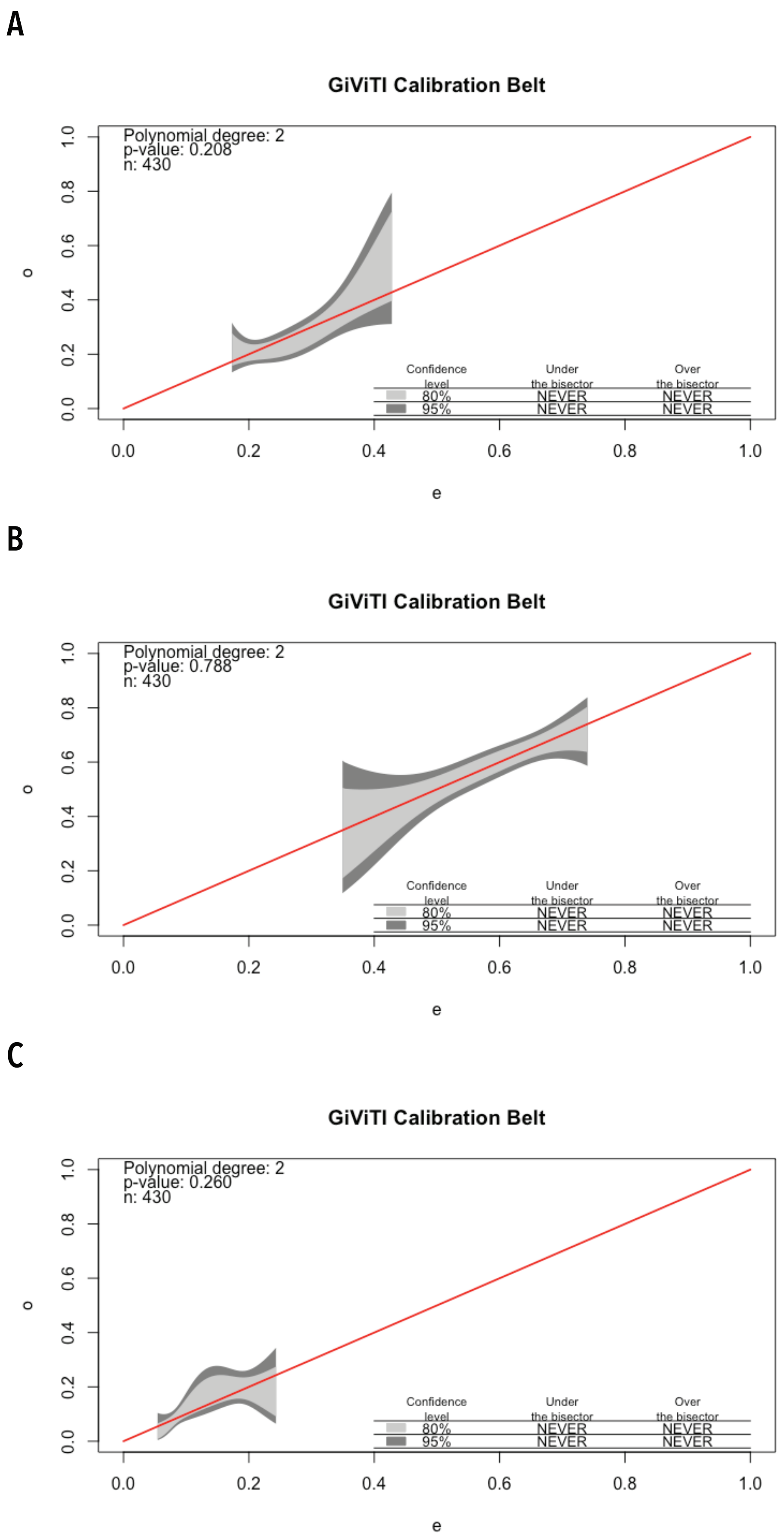

Figure S3 - Logistic Model fit. GivitICalibrationBelt-plots representing the model fit for the logistic models with 'sickness' (A), 'any symptoms' (B), and 'extreme fatigue' (C) as output variables. Related to Figure 2.

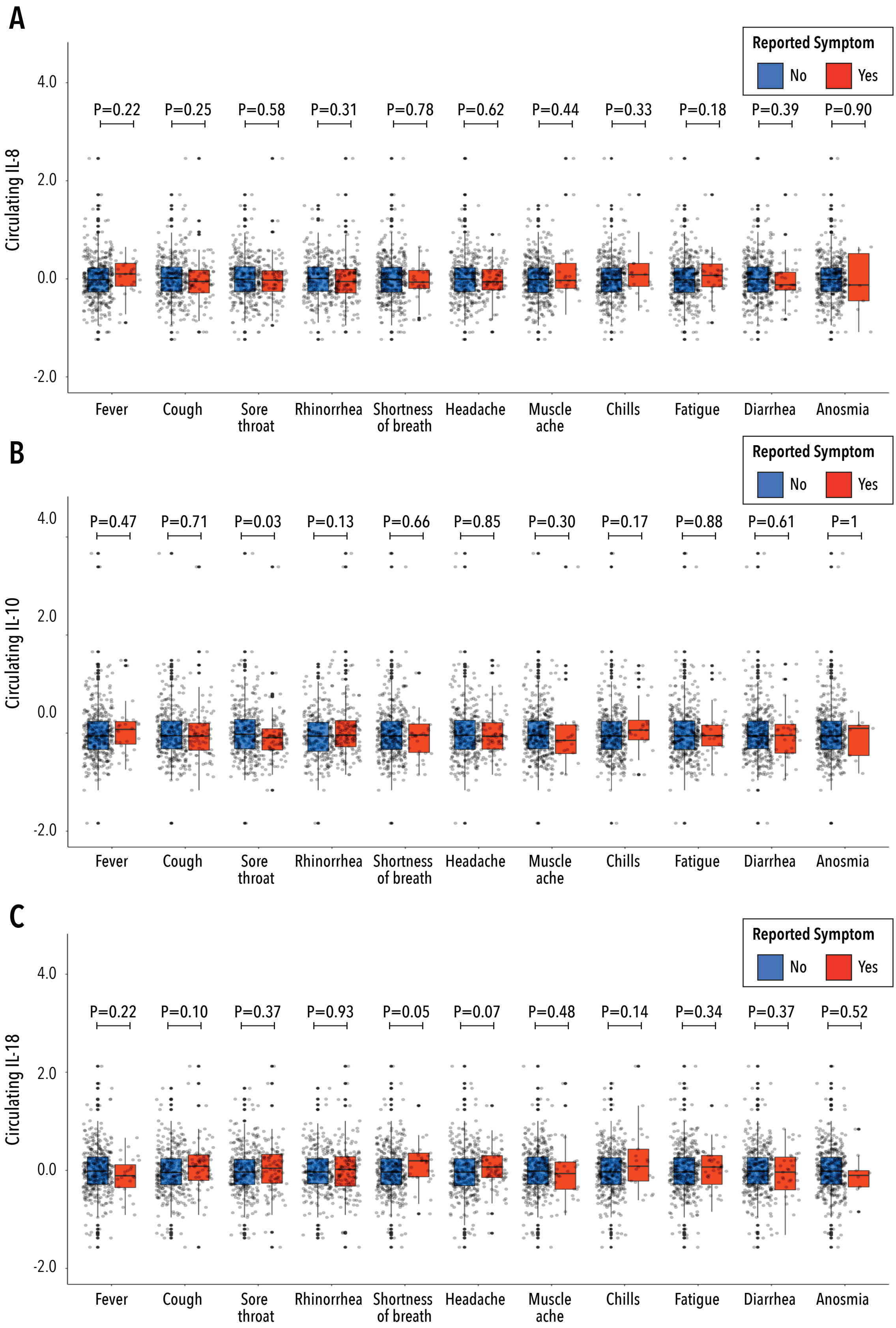

Figure S4 - Symptoms during the SARS-CoV-2 pandemic do not correlate with circulating immune parameters (n=392). Circulating concentrations of IL-8 (A), IL-10 (B) , and IL-18 (C) were normalized and measured on a log2-scale as normalized protein expression values. Differences in circulating immune parameters between individuals that reported a specific symptom were assessed using the Wilcoxon-rank sum test, and are annotated at the top of each plot. Related to Figure 3.

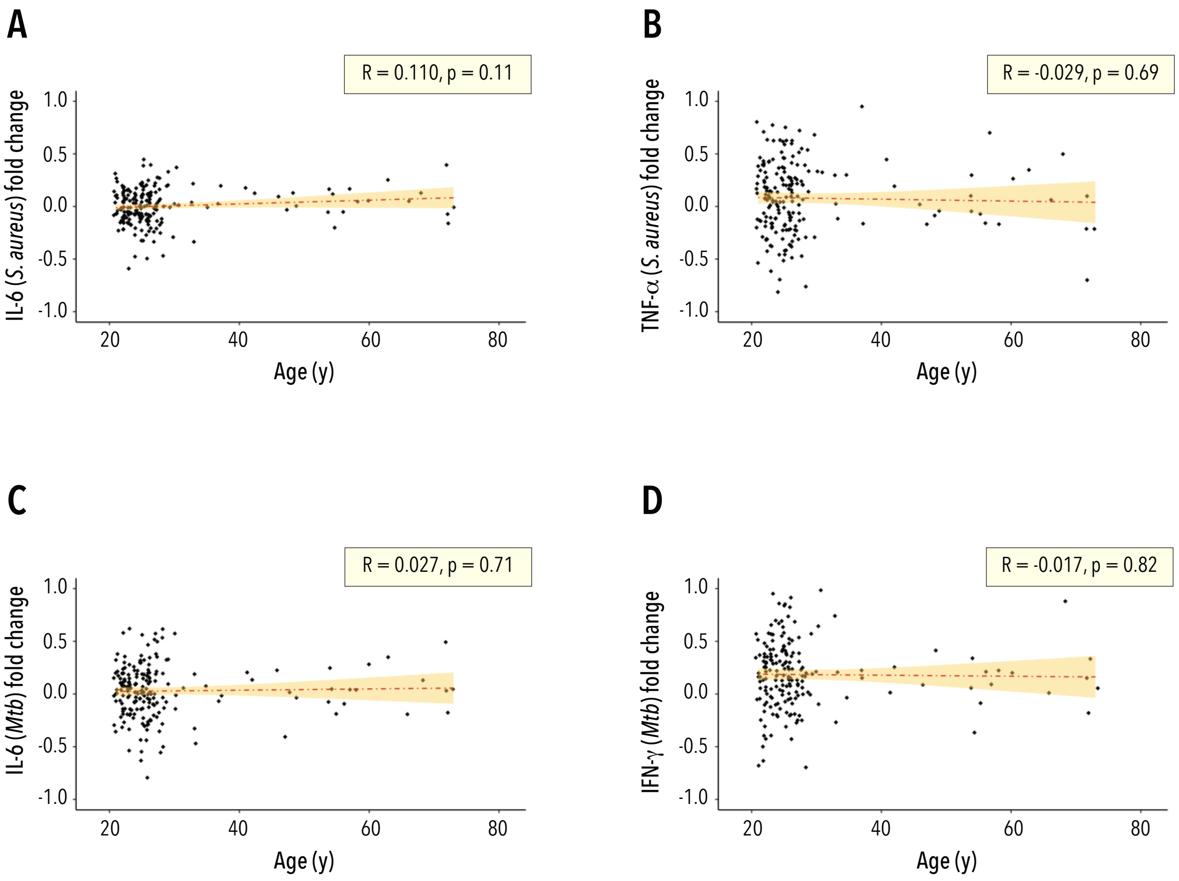

Figure S5 - Impact of age on innate memory responses induced by BCG vaccination. Log10-transformed fold changes of cytokine production upon stimulation is shown in correlation with age, with accompanying Pearson correlation and p-value. (A) IL-6 (*S. aureus*) n=195; (B) TNF-α (*S. aureus*) n=195; (C) IL-6 (*Mtb*) n=193; (D) IFN-γ (*Mtb*) n=189. Related to Figure 4.

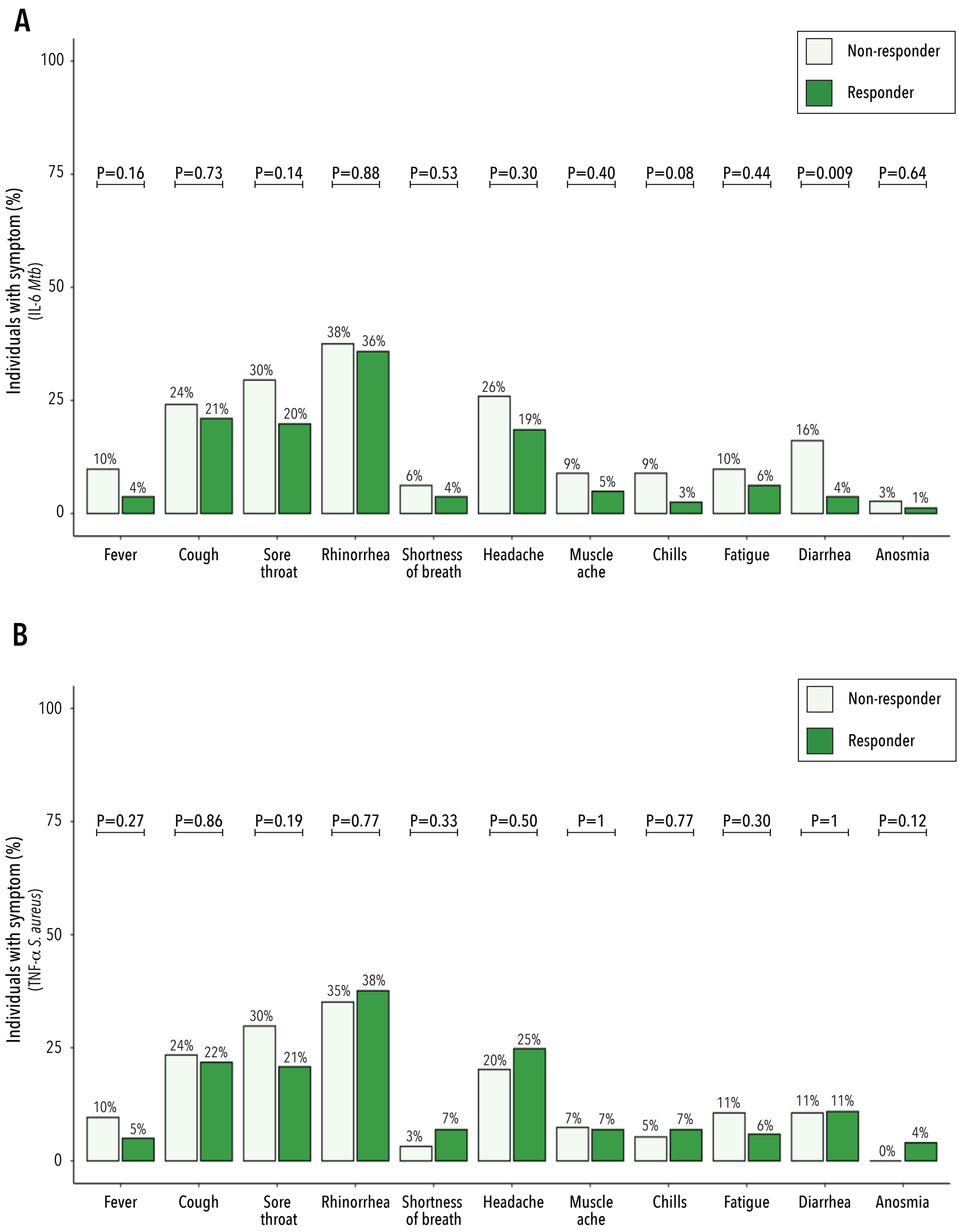

Figure S6 - Trained immunity responses correlate with self-reported symptoms during the SARS-CoV-2 pandemic. Fold change cytokine production of *Mtb*-induced IL-6 cytokine (A; n=193) and *S. aureus*-induced TNF-α cytokine (B; n=195) is shown in barplots for each reported symtpom, where individuals with a fold-change of 1.2 or higher were considered responders, and individuals with a fold change below 1.2 were considered non-responders. Percentages of non-responders (A; n=112, B; n=94) and responders (A; n=81, B; n=101) that reported symptoms are annotated above each bar, and statistical significance is annotated at the top of the plot (Fisher's Exact test). Related to Figure 4.
